# Comparative Phytochemical Profiling, Antioxidant Activity, and Chondrocyte Cytocompatibility of Water and Ethanolic Extracts from Different Organs of Golden Gardenia (*Gardinia sootepensis*)

**DOI:** 10.3390/antiox15091083

**Published:** 2026-08-28

**Authors:** Thitinun Tarathipayakul, Pattaranee Srichairatanakool, Pornpawee Sreechomphu, Vanichaya Sinpiang, Onsaya Kerdto, Peraphan Pothacharoen, Somdet Srichairatanakool, Wachiraporn Tipsuwan

**Affiliations:** 1 Department of Orthopedic, School of Medicine, University of Phayao, Phayao 56000, Thailand; thitinun.tar@up.ac.th; 2 Department of Anesthesiology, School of Medicine, University of Phayao, Phayao 56000, Thailand; pattaranee.sr@up.ac.th; 3 Research Excellence Center, School of Medicine, University of Phayao, Phayao 56000, Thailand; phronpawee0402@gmail.com (P.S.); vanichaya.sp@gmail.com (V.S.); 4 Department of Biochemistry, Faculty of Medicine, Chiang Mai University, Chiang Mai 52000, Thailand; onsaya35@gmail.com (O.K.); peraphan.p@cmu.ac.th (P.P.); 5 Division of Biochemistry, School of Medical Sciences, University of Phayao, Phayao 56000, Thailand

**Keywords:** golden gardenia, *Gardinia sootepensis*, iridoids, flavonoids, antioxidant, nutraceuticals, chondrocytes, osteoarthritis

## Abstract

*Gardenia sootepensis* Hutch. (Golden Gardinia) has received limited scientific attention despite the chemical diversity of its specialized metabolites. In this study, aqueous and 70% ethanolic preparations derived from the leaves, peel plus pulp, and seeds were systematically evaluated for extraction efficiency, total phenolic content (TPC), flavonoid content (TFC), ABTS and DPPH radical-scavenging capacity, HPLC–ESI–MS phytochemical profiles, and cytocompatibility with C28/I2 cells and primary human articular chondrocytes. The largest extract recovery was obtained from seed material, whereas the ethanolic leaf preparation contained the greatest TPC (81.08 ± 4.60 mg GAE/g), and TFC (28.36 ± 3.63 mg QE/g), and showed the strongest ABTS antioxidant activity (272.92 ± 10.95 mg TE/g). HPLC–ESI–MS analysis based on nominal-mass measurements enable the provisional annotation of 34 phytochemical features encompassing iridoids and iridoid glycosides, phenolic acids, flavonoids and their glycosides, xanthones, and benzophenone-related constituents. Overall, extraction with aqueous ethanol yielded a wider spectrum of detectable metabolites than extraction with water, while seed-derived preparations displayed the greatest chemical diversity. Under the experimental conditions and concentration range tested, viability of both immortalized C28/I2 cells and primary human articular chondrocytes remained above 80%. Collectively, these findings demonstrate that the botanical tissue selected and the extraction solvent substantially influence the phytochemical composition and antioxidant properties of *G. sootepensis*. The observed cytocompatibility provides a basis for subsequent mechanistic investigations but does not establish chondroprotective activity. Further confirmation and quantitative characterization of the provisionally annotated metabolites using high-resolution MS/MS and authentic reference standards are warranted.

## 1. Introduction

Reactive oxygen species (ROS) are generated as part of normal cellular metabolism and contribute to physiological signaling. However, when ROS production exceeds the capacity of endogenous antioxidant systems, oxidative stress develops. Persistent oxidative imbalance can induce lipid peroxidation, protein and DNA damage, mitochondrial dysfunction, and inflammatory responses [1,2,3,4]. These events contribute to the pathophysiology of several chronic and age-related diseases, including osteoarthritis (OA) [3,4,5]. In articular cartilage, elevated ROS levels can activate inflammatory pathways involving nuclear factor kappa B (NF-κB) and mitogen-activated protein kinases (MAPK), impair extracellular matrix synthesis, and enhance the production of matrix-degrading enzymes including matrix metalloproteinases (MMP) and aggrecanases [6]. Collectively, these alterations contribute to progressive cartilage deterioration and OA development [7,8,9]. Consequently, naturally derived substances capable of influencing oxidative processes while maintaining chondrocyte viability warrant investigation as potential candidates for future nutraceutical or adjunctive strategies for joint health [8,9,10,11].

Medicinal plants contain structurally diverse specialized metabolites, including phenolic acids, flavonoids, iridoids, xanthones, and coumarins, many of which exhibit antioxidant and/or anti-inflammatory properties [12,13,14,15,16]. These phytochemicals may influence redox homeostasis through several complementary mechanisms, including free-radical scavenging, transition-metal chelation, attenuation of lipid peroxidation, preservation of mitochondrial function, and modulation of endogenous antioxidant pathways such as nuclear factor erythroid 2-related factor 2/heme oxygenase-1 (Nrf2/HO-1) signaling [13,14,15]. Certain plant polyphenols have also been reported to regulate inflammatory mediators and enzymes involved in cartilage matrix degradation [6,17,18,19]. Hence, defining the chemical characteristics of botanical extracts alongside their antioxidant properties and preliminary cellular compatibility represents an important initial stage in evaluating their potential for subsequent studies related to musculoskeletal health.

*Gardenia sootepensis* Hutch., commonly referred to as golden gardenia, is a perennial member of the Rubiaceae occurring in Thailand [20]. The species is also recognized as the symbolic tree of the University of Phayao and has been consumed locally as an edible plant and used for traditional health-related purposes. Previous phytochemical studies of different *G. sootepensis* organs have reported iridoid glycosides, cycloartane triterpenes, seco-cycloartane derivatives, and other specialized metabolites [21,22,23,24,25]. Compounds isolated from this species have demonstrated a range of biological activities in experimental systems, including anti-inflammatory, anti-angiogenic, antioxidant, and cytotoxic activities [22,23,24,25]. These observations indicate that *G. sootepensis* contains chemically and biologically interesting constituents; nevertheless, the species remains considerably less characterized than the extensively investigated *G. jasminoides*.

Members of the genus *Gardenia* are particularly rich in iridoids and iridoid glycosides, together with phenolic acids, flavonoids, flavonoid glycosides, xanthones, and coumarin derivatives [21,25,26,27,28,29,30]. Representative compounds reported within this genus include geniposide, genipin, gardenoside, geniposidic acid, chlorogenic acid, caffeic acid, rutin, quercetin, and kaempferol, several of which have been associated with antioxidant, anti-inflammatory, hepatoprotective, neuroprotective, or cartilage-related biological effects [5,27,28,29,30,31,32]. Chromatographic techniques coupled with mass spectrometric detection, including high-performance liquid chromatography-electrospray ionization mass spectrometry (HPLC–ESI–MS), are useful for comparing complex phytochemical profiles and provisionally annotating metabolites recovered from different botanical tissues and extraction procedures [33,34]. Such phytochemical profiling can also facilitate the selection of candidate marker compounds for subsequent identification, quality assessment and extract standardization.

Despite increasing interest in Gardenia phytochemistry, comparative information on different anatomical parts of *G. sootepensis* remains limited. Previous investigations have focused mainly on individual constituents obtained from flowers or fruits, whereas leaves, peel plus pulp, and seeds have rarely been evaluated together. Extraction conditions represent another important variable because water and aqueous ethanol differ in their ability to recover phytochemicals with different physicochemical characteristics [23,25,33]. Accordingly, this study systematically compared water and 70% ethanolic extracts prepared from the leaves, peel plus pulp, and seeds of *G. sootepensis*. Extraction yield and phytochemical composition were examined together with chemical antioxidant activity and chondrocyte cytocompatibility. Comparative phytochemical profiles were obtained using HPLC–ESI–MS, radical-scavenging activity was assessed using ABTS and DPPH assays, and cellular compatibility was evaluated in immortalized C28/I2 cells and primary human articular chondrocytes. We hypothesized that both botanical tissue and extraction solvent would influence metabolite recovery and antioxidant performance.

## 2. Materials and Methods

### 2.1. Chemicals and Reagents

2,2′-Azino-bis(3-ethylbenzothiazoline-6-sulfonic acid (ABTS) (Product number: A1888), anhydrous aluminum chloride (Product number: 563919), *Clostridium histolyticum* collagenase (Catalogue number: C9891, 293 collagen digesting unit (CDU)/mg solid), Dulbecco’s modified Eagle’s medium (DMEM) (Product number: D6429), dimethyl sulfoxide (DMSO) (Product number: D2650), 2,2-diphenyl-1-picrylhydrazyl (DPPH) (Product number: D9132), fetal bovine serum (FBS) (Product number: F2442), formic acid (Product number: 695076), [3-(4,5-dimethylthiazol-2-yl)-2,5-diphenyltetrazolium bromide] (MTT) (Product number: 475989), phosphate-buffered saline (PBS) pH 7.4 (Product number: P4474), quercetin (Q) (Product number: Q4951), sodium carbonate (Product number: S2127), and gallic acid (GA) (Product number: G7384) were obtained from Sigma-Aldrich Chemicals Company Limited, Saint Louis, MO, USA. Folin–Ciocalteu reagent (Reagent Code: 251567) was bought from Panreac Química S.L.U., an ITW Company, Castellar del Vallès, Barcelona, Spain. Accordingly, 6-Hydroxy-2,5,7,8-tetramethylchromane-2-carboxylic acid (Trolox) (Catalogue number: 648471), potassium acetate (Catalogue number: 104820), and potassium persulfate (Catalogue number: 216224) were purchased from Merck-Millipore Company, KGaA, Darmstadt, Germany. Penicillin–streptomycin and 1× trypsin–EDTA with phenol red (Catalogue number: 25200-056) were purchased from Gibco, Thermo Fisher Scientific, Waltham, MA, USA. All solvents, including acetonitrile, ethanol, and methanol, were of HPLC or the highest-pure grade.

### 2.2. Ethics Approval and Informed Consent

The study protocol received approval from the Human Research Ethics Committee of the University of Phayao, Thailand (HREC-UP-HSST 1.2/048/69; 12 March 2026). Human cartilage used for primary chondrocyte isolation was collected in accordance with the ethical standard established in the Declaration of Helsinki and the International Council for Harmonisation Guidelines for Good Clinical Practice (ICH-GCP) principles. All donors provided written informed consent before their samples were collected, including consent for the specimens to be used for research purposes.

### 2.3. Plants and Extract Preparations

**Figure 1:**
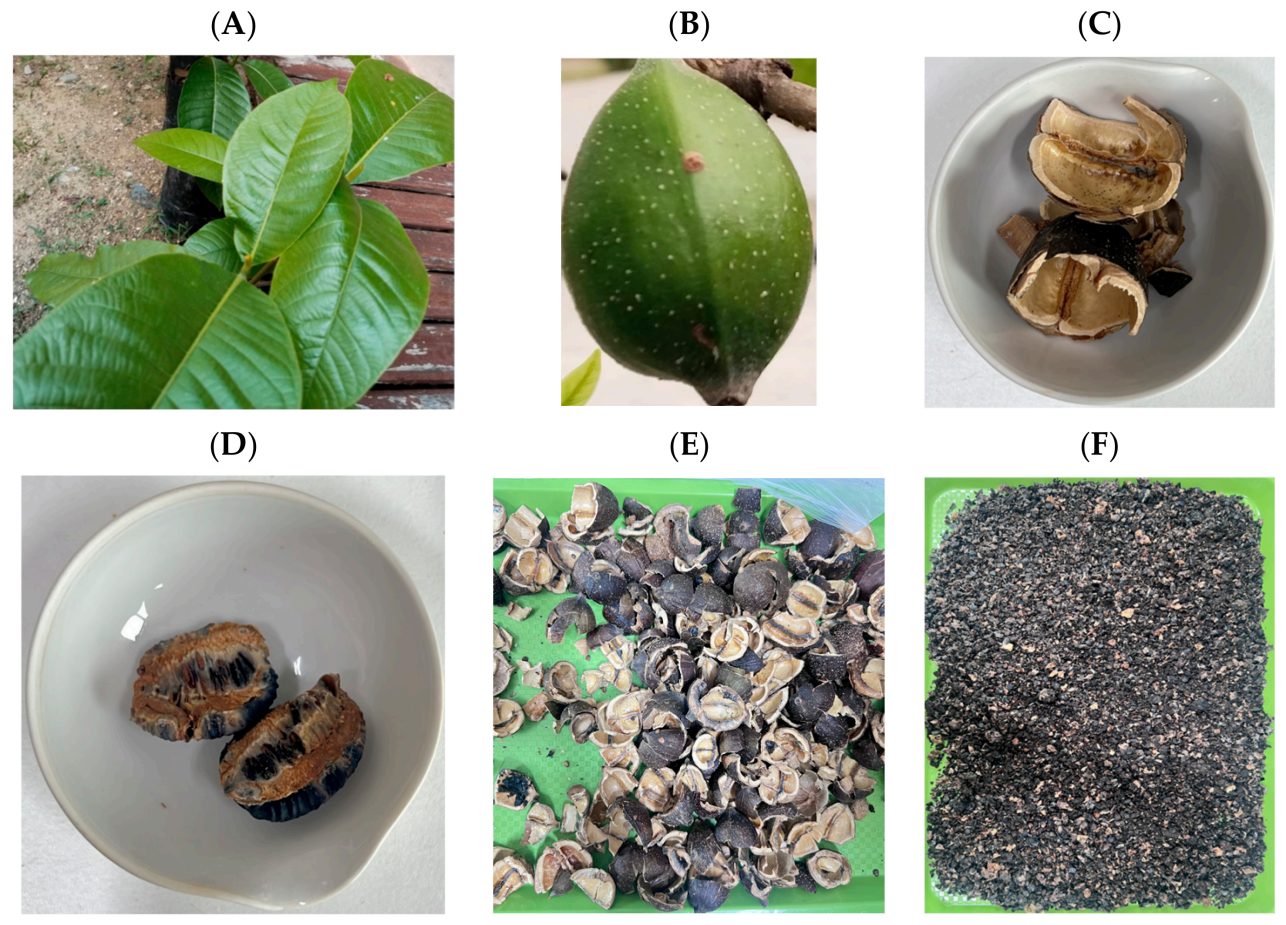
Appearance of golden gardenia (*Gardenia sootepensis* Hutch.) plant materials. (**A**) Leaves; (**B**) whole fruit; (**C**) fresh fruit showing peels, pulp, and seeds; (**D**) dried fruit components; (**E**) dried peels; and (**F**) dried seeds.

A specimen of golden gardenia (*G. sootepensis* Hutch.), including leaves and fruits, was kindly provided by Golden-T Siam Company Limited (Pai, Mae Hong Son, Thailand). Botanical identity was confirmed by the Department of Pharmaceutical Sciences, Faculty of Pharmacy, Chiang Mai University, when a voucher specimen was deposited under accession number 0023433. Fresh leaves were collected on 22 October 2025 (Figure 1A), and fruits were collected on 11 January 2026 (Figure 1B). Fresh leaves (L; 500 g) were rinsed twice with 2 L of clean tap water, and subsequently oven-dried at 60 °C for 24–48 h. The dried material was then pulverized to a powder and separated into portions for extraction with either water or ethanol. The fruit material was separated into pulp with peels (PuPe) and seeds (S) fractions (Figure 1C). The two fruit fractions (500 g fresh weight each) were individually dehydrated in a hot-air oven (60 °C) for 24–48 h. Following drying, the pulp-plus-peel material (Figure 1D,E) and seeds (Figure 1F) were separately milled into fine powders. Each powdered preparation was then portioned for subsequent extraction using either hot water or 70% ethanol.

Water extracts of golden gardenia leaves (WEGL), peels + pulp (WEGPuPe) and seeds (WEGS), and their ethanolic extracts (EEGL, EEGPuPe, and EEGS) were prepared using the procedure described by Paradee et. al. [35]. In the preparation stage, the leaf, peel + pulp, and seed powder (250 g each) were extracted with deionized water (DI) (1.25 L) at 70 °C for 10 min and filtered through a food-grade nylon screen (200 mesh; 100 μm). Separately, a 250 g portion of the powdered materials was immersed in 1.25 L of 70% (*v*/*v*) ethanol and extracted at ambient temperature for 24 h, followed by passing through a nylon sieve. The recovered extracts were centrifuged at 8000 rpm for 15 min, after which the resulting supernatants were filtered through solvent-resistant cellulose filter paper (Whatman Grade 4, Product number WHA1004125, Maidstone, UK). Ethanol was subsequently eliminated from the filtrates by rotary evaporation at 50 °C using a Drawell rotatory evaporator (Drawell International Technology Limited Company, Chongqing, China). Afterwards, the remaining water was removed by lyophilization until the extracts were completely dried. The resulting extract powders were weighed, transferred to plastic containers, and stored at −20 °C until further analysis. Extraction yield was subsequently determined by expressing the mass of the recovered dried extract relative to the initial dry mass of the plant material.

### 2.4. Phytochemical Composition Assessment

#### 2.4.1. Total Phenolic Content

TPC in WEGL, WEGS, EEGL, and EEGS was quantified by the Folin–Ciocalteu assay. Briefly, a 20 µL aliquot of DI (blank), GA standard (12.5–400 µg/mL), or approximately diluted extracts was combined with 100 µL of Folin–Ciocalteu reagent prepared at 10% (*v*/*v*). The reaction mixtures were kept at room temperature in the dark for 3 min, followed by the addition of 80 μL of 7.5% (*w*/*v*) sodium carbonate. The reaction mixtures were thoroughly mixed and maintained at room temperature in the dark for 30 min. Optical density (OD) was subsequently recorded at 750 nm against the corresponding blank using a double-beam ultraviolet/visible (UV/VIS) spectrophotometer (Model 1900, Shimadzu Corporation, Kyoto, Japan). TPC was calculated from the calibration curve generated with GA as the reference standard and reported as milligram gallic acid equivalents (GAE)/g extract.

#### 2.4.2. Total Flavonoid Content

The total flavonoid content (TFC) of the extracts was quantified using the aluminum chloride colorimetric assay [36]. Briefly, 20 µL aliquots of DI (blank), standard Q (12.5–400 µg/mL), and diluted extract solutions ranging from 0.0781 to 10 μg/mL were combined with 80 µL of 0.5% (*w*/*v*) aluminum chloride solution and 100 µL of 40 mM potassium acetate solution. The reaction mixtures were incubated at room temperature in the dark for 30 min, after which OD values were recorded at 405 nm against the blank using a UV/VIS spectrophotometer. TFC was calculated from the quercetin standard calibration curve and expressed as milligram quercetin equivalents (QE)/g extract.

#### 2.4.3. Phytochemical Profiling of Phenolic Constituents

The polar phenolic compounds of the extracts were profiled using the HPLC-ESI-MS method [37]. Chromatographic separation was performed using an Agilent 1100 Series platform (Agilent Technologies, Deutschland GmbH, Waldbronn, Germany) equipped with a G1311A quaternary pump, an online G1322A vacuum degasser, a G1313A autosampler, a G1316A thermostated column compartment and a G1315A photodiode array (PDA) detector. The PDA outlet was connected via a 1:1 flow splitter to the atmospheric pressure ESI interface of an Agilent 1100 LC/MSD SL MS detector (Agilent Technologies, Palo Alto, CA, USA). Separation was achieved on a LiChroCART RP-18e column (150 mm × 4.6 mm, 5 µm particle size; Purospher STAR, Merck, Darmstadt, Germany), with the column temperature maintained at 40 °C.

For sample preparation, 10 mg of each extract was dissolved in 1 mL of methanol and passed through a polytetrafluoroethylene Acrodisc syringe membrane filter (0.45 μm pore size, 13 mm diameter; Merck Millipore Limited Company, Burlington, MA, USA) to eliminate suspended particulates. A 10 μL aliquot of the filtered sample was subsequently introduced into the HPLC system. Chromatographic separation employed acetonitrile as mobile phase A and 10 mM formate buffer (pH 4.0) as mobile phase B. The gradient program was as follows: 0–5 min, 100% B; 5–10 min, a linear increase from 0 to 20% A; 10–20 min, 20% A; and 20–60 min, a linear increase from 20 to 40% A. The mobile-phase flow rate was maintained at 1.0 mL/min, and PDA detection was performed at 270 nm. Authentic standards of gallic acid (GA), catechins, rutin, isoquercetin, hydroquinine, eriodictyol, apigenin, and kaempferol were analyzed when available to assist with compound identification. Mass spectrometric analysis was conducted using positive-mode electrospray ionization, with spectra collected over an *m*/*z* range of 100–700. For the single-quadrupole MS system, the ESI energy was maintained at 70 eV, while the ion-source and interface temperatures were set at 150 and 230 °C, respectively. Nitrogen served as the nebulizing, drying, and collision gas. The capillary temperature was maintained at 320 °C, with a nebulizer pressure of 60 psi and a drying-gas flow rate of 13 L/min. The capillary voltages were 3500 V in positive mode and 150 V in negative mode. The oven-temperature program began at 80 °C with a 3 min hold, followed by an increase to 110 °C at 10 °C/min and a 5 min hold. The temperature was subsequently raised to 190 °C and maintained for 3 min, increased to 220 °C at 10 °C/min and held for 4 min, and finally elevated to 280 °C at 15 °C/min with a 13 min hold. Automatic mass calibration was performed using an external calibration solution (ESI-L Low Concentration Tuning Mix; Agilent Calibration Solution B). The reported limit of detection, limit of quantification, and recovery range were 0.5 mg/kg, 1.20 mg/kg, and 70–110%, respectively. Chromatographic and mass-spectral data processing, molecular-formula prediction, and exact-mass calculations were carried out using MassHunter software version B.04.00, build 4.0.479.0 (Agilent Technologies). For phytochemical profiling, the lyophilized extracts were reconstituted in 1.0 mL of an equal-volume mixture of mobile phases A and B before HPLC–MS analysis. Chromatographic features were evaluated according to their retention times and observed *m*/*z* values. Metabolite assignments were considered provisional and were made by comparison with available authentic reference standards and previously reported phenolic constituents.

### 2.5. Investigation for Toxicity of Extracts in Chondrocyte Cultures

#### 2.5.1. Human Chondrocyte Cell Line

The immortalized human chondrocyte cell line C28/I2 was provided by Dr. Peraphan Pothacharoen, Department of Biochemistry, Faculty of Medicine, Chiang Mai University. C28/I2 cells were maintained in DMEM containing 10% (*v*/*v*) FBS, 100 U/mL penicillin and 100 μg/mL streptomycin at 37 °C in a 5% CO_2_ incubator [38]. The culture medium was renewed every 2–3 d, with cell morphology monitored regularly. Passaging was performed when the cell density reached approximately 70–80% confluence. The medium was removed, washed once with PBS, and cells were detached by adding 1× trypsin-EDTA (0.25%), followed by incubation at 37 °C for approximately 2–5 min. The flask was then gently tapped to release the cells. Complete medium was subsequently added to neutralize trypsin activity. The cell suspension was pelleted by centrifugation at 1200 rpm for 5 min. The centrifuged cells were then transferred to the new medium and further cultured.

#### 2.5.2. Human Primary Cartilage Cells

##### Specimen Collection

Cartilage tissue samples, which were free of bone and soft tissue, were surgically excised from the knees of 6 patients aged 45–68 y (60.2 ± 8.8 y, 1 male and 5 females) who had undergone knee arthroplasty (Table 1) by a well-trained orthopedic surgeon, Dr. Thitinun Tarathipayakul, MD, at the University of Phayao Hospital, School of Medicine, University of Phayao. All surgical procedures were performed following ethical committee approval, and written informed consent in Thai was obtained from each participant before the procedure.

All experimental procedures were conducted with the applicable guidelines and regulatory requirements. Dissected cartilage tissue samples were transferred into a low-oxygen (hypoxia) chamber and immediately placed in an ice box (4–5 °C, 2–3 h transportation). The specimens were subsequently transported to the Tissue Engineering Laboratory, Department of Biochemistry, Faculty of Medicine, Chiang Mai University, for further experiments.

##### Isolation and Cultivation of Chondrocytes

Primary human articular chondrocytes (HAC) were obtained from knee cartilage specimens and expanded ex vivo in DMEM supplemented with FBS. In this procedure, cartilage tissue samples were minced into small fragments, finely ground, and digested with 0.2% (*w*/*v*) collagenase solution (586,000 CDU/L) with 2 µg/mL of actinomycin D for 24 h at 37 °C with gentle agitation while maintaining sterile conditions to release HAC into suspension. The recovered chondrocytes were then washed and seeded into tissue culture flasks containing DMEM with 10% (*v*/*v*) FBS and penicillin-streptomycin, where they adhered and subsequently proliferated in the monolayer culture. The resulting digest was subsequently passed through a 70 µm cell strainer, after which the recovered cells were washed with buffer solution [39]. Under these conditions, HAC can be expanded over multiple passages (P0-P5) while maintaining a chondrogenic phenotype.

#### 2.5.3. MTT-Based Cytotoxicity Assessment

Prior to cell treatment, the aqueous extracts were freshly reconstituted in sterile DI, while the ethanolic extracts were freshly prepared by dissolving them in sterile 1% (*v*/*v*) DMSO, and the extract solutions were sterilized by filtration through a 0.22 µm pore-size membrane filter. In the assay, 100 μL of C28/I2 cells or HAC (1 × 10^4^/well) were treated with 100 μL of the plant extracts at concentrations ranging from 7.1825 to 500 μg/mL, cultured in a CO_2_ incubator for either 24 or 48 h at 37 °C, followed by assessment using the MTT test. The MTT assay was employed as an indirect indicator of cell viability by assessing mitochondrial metabolic activity. Briefly, 100 μL of MTT solution (0.5 mg/mL) was added to each well, followed by incubation at 37 °C for 4 h. The culture media were subsequently removed, and 1% DMSO solution (100 µL/well) was added to solubilize the purple formazan crystals. OD value was recorded at 570 nm against a reagent blank [36]. and cell viability was subsequently calculated and expressed as a percentage relative to the corresponding control.

### 2.6. Evaluation of Antioxidant Activity

#### 2.6.1. ABTS Method

The antioxidant activity (AA) of the extracts was assessed using the ABTS radical-cation decolorization method [40]. The ABTS radical cation (ABTS^•+^) was produced by combining 7 mM ABTS with 2.45 mM potassium persulfate. The prepared radical solution was subsequently diluted with PBS (pH 7.4) to an OD of 0.70 at 734 nm. For the assay, 20 μL of either Trolox standard (0.0156–1 mg/mL) or extract solution was combined with 1.0 mL of the ABTS^•+^ working solution. Following incubation at room temperature for 6 min, OD was measured at 734 nm against a reagent blank using a UV–Vis spectrophotometer. The percentage of ABTS^•+^ inhibition was calculated according to Equation (1)

% Inhibition of ABTS^•+^ generation = [(OD_PBS_ − OD_sample_)/OD_PBS_] × 100 (1)

where OD_PBS_ = ABTS^•+^ solution was treated with PBS and OD_sample_ = ABTS^•+^ solution was treated with Trolox or extracts. A dose–response inhibitory curve of ABTS^•+^ generation was plotted by contrasting the compound concentrations (*x*-axis) against the percentages of the inhibition (*y*-axis). Half-maximal inhibitory concentration (IC_50_) values of the ABTS^•+^ generation were then determined from the curves.

#### 2.6.2. DPPH Method

DPPH radical (DPPH^•^) scavenging activity was assayed using a reference [41]. For each reaction, 20 µL of extracts or Trolox standard was mixed with 180 µL of 0.4 mM DPPH^•^ solution and incubated under the assay conditions. The decrease in OD at 517 nm relative to the control represented radical inhibition. Similarly, the DPPH^•^ scavenging activity was calculated using Equation (2)

% Inhibition of DPPH^•^ generation = [(OD_PBS_ − OD_sample_)/OD_PBS_] × 100. (2)

The results were presented as percentages of inhibition of ABTS^•+^ and DPPH^•^ generation by Trolox or the extracts together with their IC_50_ and AA values.

### 2.7. Statistical Evaluation

Data are presented as the mean ± standard deviation (SD) or ± standard error of the mean (SEM), as specified for each experiment. Statistical analyses were conducted using SPSS Inc. Software version 21 (IBM Corporation, Chicago, IL, USA). Differences involving multiple groups were examined by one-way or two-way analysis of variance (ANOVA), as appropriate, followed byTukey’s post hoc multiple-comparison test. For comparisons between two groups, Student’s *t*-test was applied where applicable. *p*-value < 0.05 was considered statistically significant.

## 3. Results

### 3.1. Appearance and Extraction Yields of Water and Ethanolic Extracts

**Figure 2:**
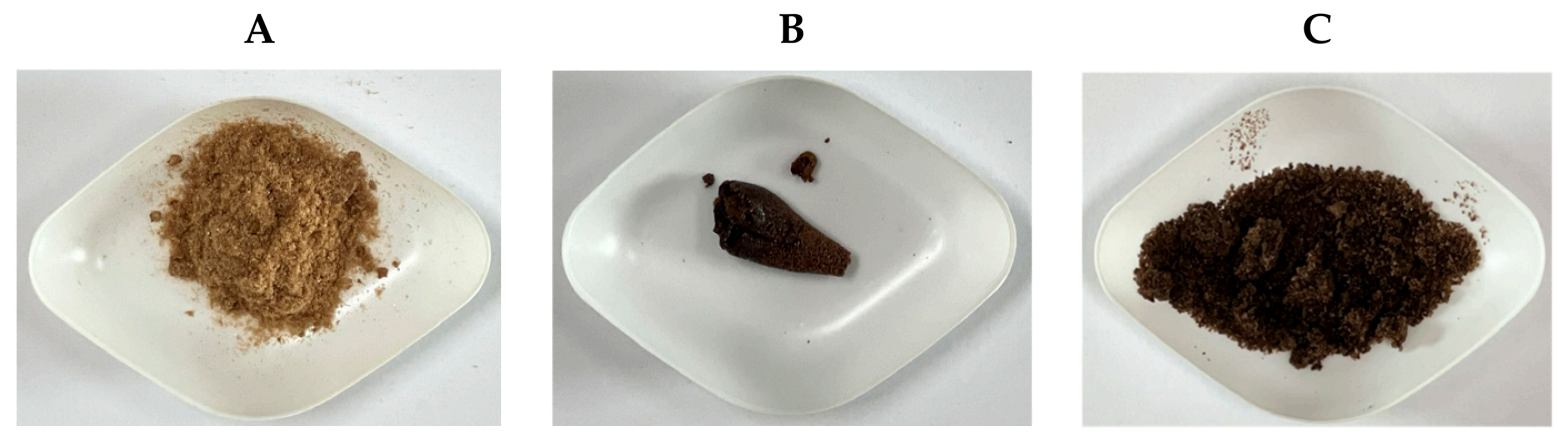

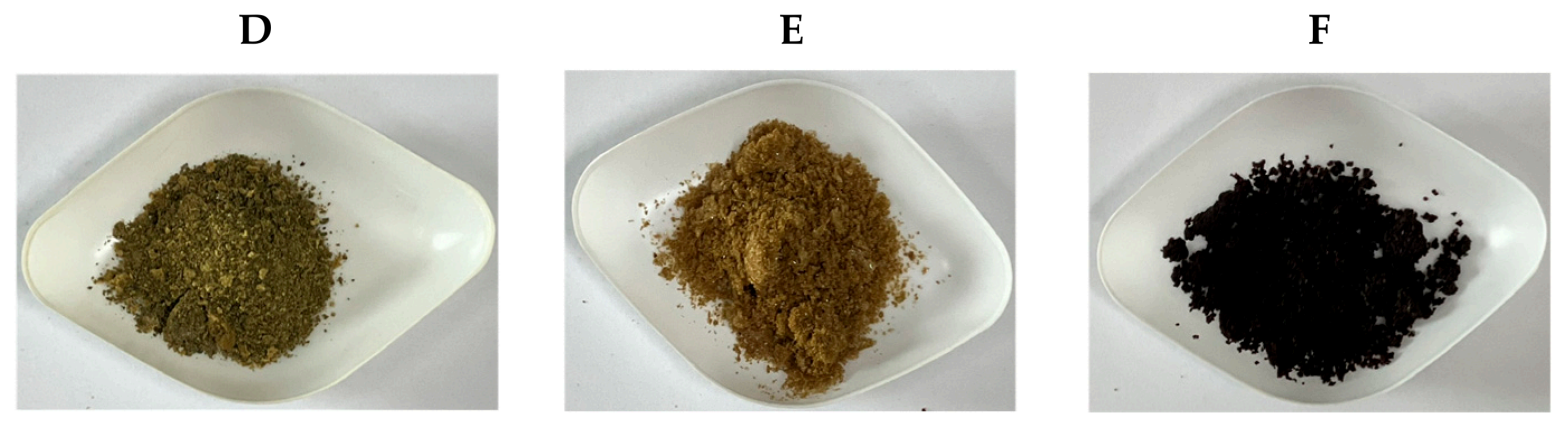
Appearance of golden gardenia (*Gardenia sootepensis* Hutch.) plant extracts. (**A**) Water extract of leaves; (**B**) water extract of peels and pulp; (**C**) water extract of seeds; (**D**) ethanolic extract of leaves; (**E**) ethanolic extract of peels and pulp; and (**F**) ethanolic extract of seeds.

Figure 2 summarizes the visual characteristics of the golden gardenia (*G. sootepensis* Hutch.) materials and the resulting preparations. The water and ethanol-derived extracts differed in appearance according to both tissue sources and solvent, providing a qualitative indication that extraction conditions recovered different mixtures of constituents. These observations were subsequently examined quantitatively through extraction yield and chemical analyses.

Extraction efficiency depended on both plant organs and solvent (Table 2). Based on a dry-weight basis, WEGS produced the greatest yield (116.17 mg/g DW, 11.62%), followed by EEGS (76.41 mg/g DW, 7.64%). Lower recoveries were obtained from the leaf and peel plus pulp preparations. Thus, seed materials yielded the largest amounts of dried extract under the conditions used. Whereas the relative performance of water and 70% ethanol varied with plant part.

### 3.2. Chemical Composition Analysis

#### 3.2.1. Quantification of Total Phenolic and Flavonoid Contents

Phenolic and flavonoid contents differed among the six preparations (Table 3). EEGL contained the greatest TPC (81.08 ± 4.60 mg GAE/g), with WEGL next highest (71.72 ± 4.06 mg GAE/g). Except for the comparison between EEGS and WEGS, TPC differed significantly among extracts (*p* < 0.05). EEGL also had the largest TFC (28.36 ± 3.63 mg QE/g), whereas WEGS contained 24.38 ± 0.08 mg QE/g. EEGS showed the lowest TPC (0.43 ± 0.30 mg GAE/g) and a comparatively low TFC (2.47 ± 0.47 mg QE/g). For TFC, EEGL and WEGL did not differ significantly, and no significant differences were detected among EEGS, WEGPuPe, and WEGS; the remaining pairwise comparisons were significant (*p* < 0.05).

Collectively, the leaf-derived extracts contained considerably higher levels of phenolic and flavonoid constituents than the peel-plus-pulp and seed preparations, with the greatest enrichment observed following ethanol extraction. Notably, the markedly lower TPC of EEGS, when compared with WEGS, was contrasted with the phytochemical features detected by HPLC–ESI–MS, as has been presented below.

#### 3.2.2. Provisional HPLC-ESI-MS Annotation of Golden Gardenia Extracts

**Figure 3:**
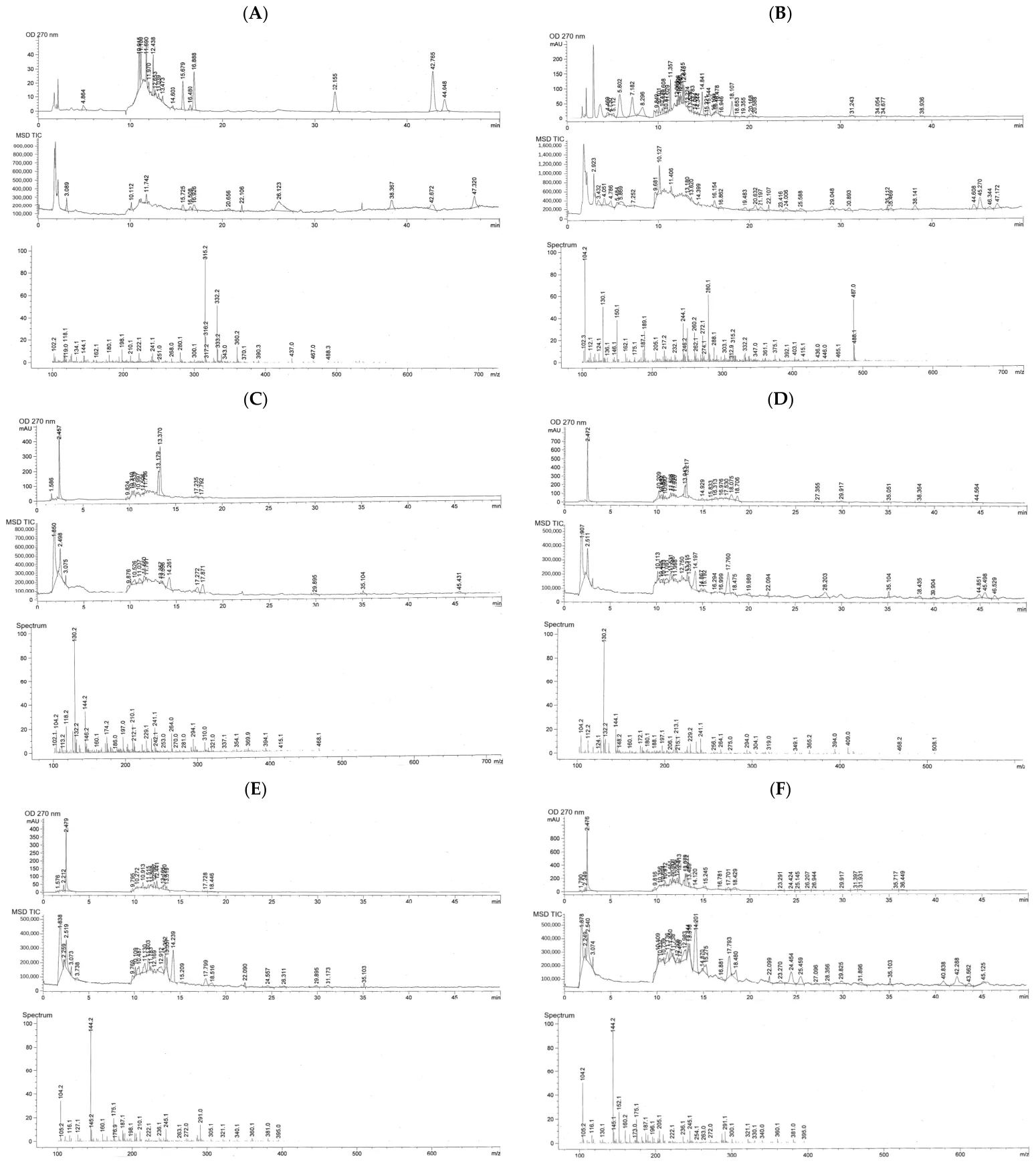
HPLC-ESI-MS profiles of polar phenolic compounds of water and ethanolic extracts prepared from leaves (**A**,**B**), pulp plus peel (**C**,**D**), and seeds (**E**,**F**) of golden gardenia (*Gardenia sootepensis* Hutch.).

An HPLC-ESI-MS (Agilent 1100 LC/MSD SL single quadrupole) machine was used to identify polar phenolic compounds in six extracts; however, this platform supplied nominal-mass data without accurate-mass or diagnostic tandem-MS information. Therefore, the reported identities are provisional and were assigned from retention behavior, nominal *m*/*z*, authentic standards when available, and literature evidence, corresponding mainly to MSI Levels 3–4. Figure 3 compares the HPLC–ESI–MS profiles of water and ethanolic extracts prepared from leaves (A–B), pulp plus peel (C–D), and seeds (E–F). The chromatograms demonstrate chemically complex profiles in all six extracts, with numerous signals distributed over the chromatographic run. Importantly, the overall occurrence of many corresponding features across the six extracts indicates that the different plant organs share a substantial common phytochemical composition. The early-to-intermediate retention-time region contains numerous signals provisionally associated with iridoids and iridoid glycosides, whereas later regions include phenolic acids, flavonoids/flavonoid glycosides, xanthones, and other conjugated constituents. Nonetheless, the profiles should not be interpreted simply from peak height as demonstrating that one extract contains quantitatively more of a particular compound. Unless peak areas were integrated, normalized, calibrated, and statistically compared, Figure 3 primarily provides qualitative/semi-qualitative evidence of compositional differences. In particular, the ethanolic extracts contain several additional late-eluting features that are absent from their corresponding water extracts. This agrees closely with Table 4 and Table S7 and indicates that ethanol recovered a somewhat broader range of relatively less polar or more structurally complex phytochemicals.

Table 4 provisionally annotates 34 phytochemical features across the six extracts. The proposed constituents span several chemical families, particularly iridoids/iridoid glycosides, phenolic acids, flavonoids and flavonoid glycosides, xanthones, a benzophenone-related compound, and higher-molecular-weight conjugates. One of the strongest findings is the presence of a broad iridoid signature. Geniposide and gardenoside were detected at approximately 9.82 and 10.11 min, respectively, together with genipin and several other proposed iridoid-related compounds. These observations indicated that iridoid-associated metabolites constituted a prominent component of the phytochemical profile of *G. sootepensis*. The extracts also contained a substantial phenolic/flavonoid component. The annotated features include quinic acid, chlorogenic acid, caffeic acid, ferulic acid, rutin-related compounds, quercetin and kaempferol glycosides, quercetin, kaempferol, and isorhamnetin. Therefore, rather than being dominated by a single chemical class, the extracts appear to contain a multicomponent phytochemical matrix, comprising both iridoid-type metabolites and several classes of phenolic constituents. This chemical diversity could provide a reasonable phytochemical basis for antioxidant activity observed elsewhere in the study, although the HPLC–ESI–MS data alone cannot establish which compounds are responsible for that biological activity. Particularly important, peak 29, provisionally assigned as an acetylated hyperoside-related flavonoid glycoside, was detected only in EEGL, EEGPuPe, and EEGS—not in the corresponding water extracts. Likewise, peak 33, provisionally assigned as an iridoid–phenolic conjugate, and peak 34, a high-molecular-weight polyphenolic conjugate, occurred exclusively in the three ethanolic extracts. This provides direct evidence of solvent-dependent extraction selectivity. Ethanol appears to recover certain structurally complex or relatively less water-soluble phytochemical constituents that are not detected in the aqueous extracts under the analytical conditions used.

In comparison among plant organs, most of the provisionally annotated compounds are shared among leaves, pulp plus peel, and seeds. For example, geniposide, gardenoside, genipin, quinic acid, chlorogenic acid, caffeic acid, several flavonoid glycosides, quercetin, kaempferol, isorhamnetin, xanthone-related compounds, and the benzophenone-related feature were detected across all six preparations. Therefore, the data suggest that the plant organ has less influence on simple presence/absence than solvent extraction for many of the detected metabolites. However, there is an organ-specific difference in the very early features. Gluconic acid and dehydroascorbic acid, corresponding to peaks 2 and 3 in the current annotation, were detected in leaf and seed extracts but were not detected in either pulp-plus-peel extract. Thus, the chemical differentiation appears to involve both organ-dependent differences in selected early/polar features and solvent-dependent differences in later/high-molecular-weight constituents.

Moreover, Tables S1–S6 provide the individual phytochemical profiles underlying the combined presentation in Table 4. For examples. WEGL contains the major iridoid, phenolic acid, flavonoid, xanthone, and benzophenone-related features but lacks the ethanol-associated peaks 29, 33, and 34. The table includes geniposide, gardenoside, genipin, chlorogenic acid, caffeic acid, flavonoid glycosides, quercetin, kaempferol, and isorhamnetin (Table S1). EEGL shows a broader profile than WEGL, including peaks 29, 33, and 34. This supports increased chemical coverage by ethanol rather than simply extraction of an entirely different phytochemical system (Table S2). WEGPuPe differs somewhat from WEGL and WEGS because peaks 2 and 3 are absent. Nevertheless, the majority of the iridoid, phenolic acid, and flavonoid features remain present (Table S3). EEGPuPe shows the ethanol-associated compounds, including acetylated hyperosides, the proposed iridoid–phenolic conjugate, and the high-molecular-weight polyphenolic conjugate (Table S4). WEGS resembles the aqueous leaf extract and contains the early features that are absent from the pulp-plus-peel preparations, including the proposed gluconic acid and dehydroascorbic acid features (Table S5). Finally, EEGS displays one of the broad ethanol-associated profiles and contains peaks 29, 33, and 34 in addition to the common phytochemical core (Table S6). Consequently, the results of Tables S1–S6 collectively support the interpretation that ethanol broadens the detectable phytochemical spectrum, while the major iridoid–phenolic–flavonoid framework is conserved among the three investigated organs. Table S7 is the most useful for comparative interpretation because it summarizes presence/absence across all six extracts. Collectively, these results demonstrate that differences in solvent polarity appear to influence the qualitative phytochemical profile more clearly than plant organ for the later-eluting specialized metabolites, whereas most major compounds are broadly distributed throughout the investigated organs.

### 3.3. Effects of Water and Ethanolic Extracts on Human Chondrocyte Viability

#### 3.3.1. Viability of C28/I2 Cells Following Treatment with the Extracts

**Figure 4:**
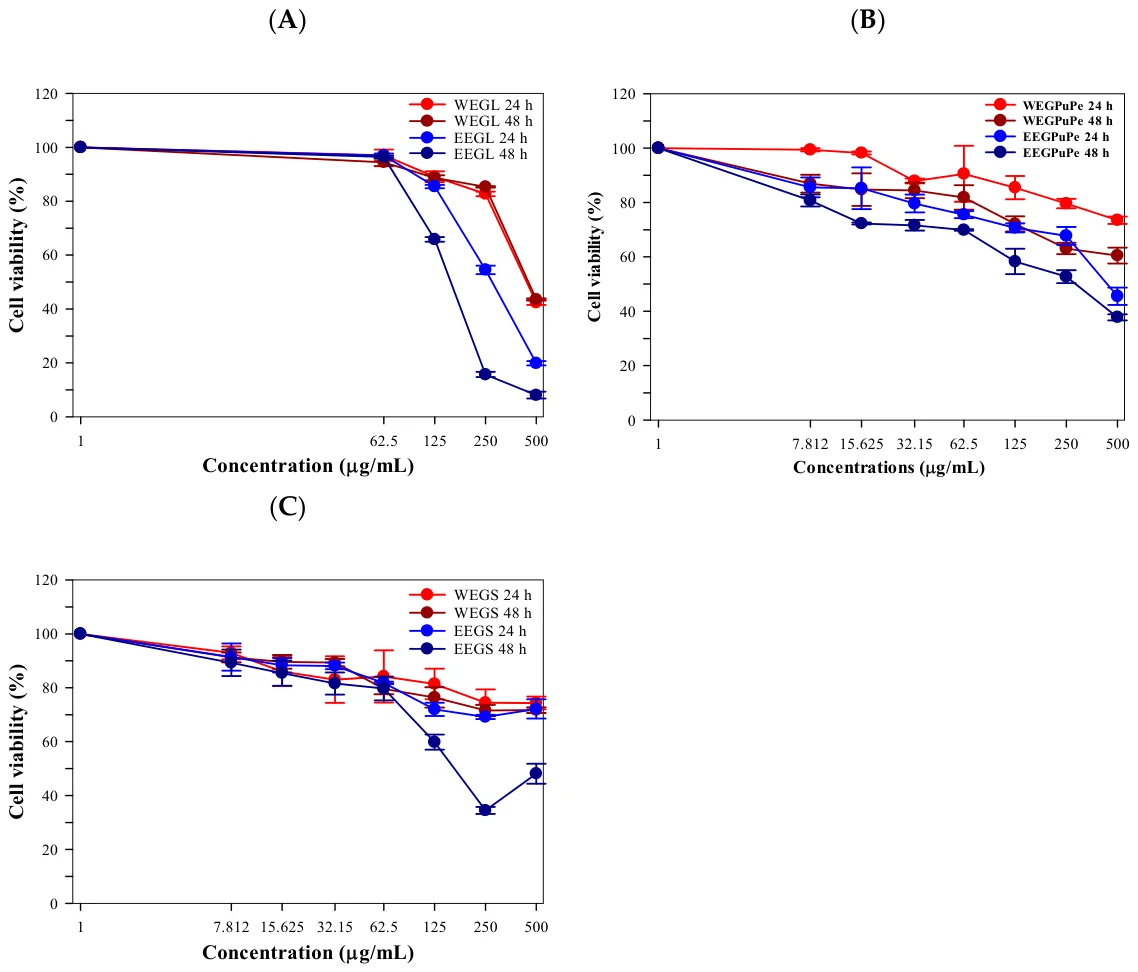
Viability of C28/I2 human chondrocytes following treatment with (**A**) water and ethanolic extracts of golden gardenia leaves (WEGL and EEGL), (**B**) water and ethanolic extracts of golden gardenia pulp plus peel (WEGPuPe and EEGPuPe), and (**C**) water and ethanolic extracts of golden gardenia seeds (WEGS and EEGS) for 24 and 48 h. Results are presented as the mean ± SD obtained from three independent experiments, each performed in triplicate.

Viability of C28/I2 human chondrocytes following treatment with WEGL and EEGL (7.8125–500 μg/mL) for 24 (or 48) h, as assessed by the colorimetric MTT assay (Figure 4A). The WEGL-treated cells exhibited relatively stable cell viability over the concentration range tested, with no marked concentration-dependent reduction in mitochondrial metabolic activity. Likewise, cell viability following EEGL exposure remained comparable to that observed in the untreated control, although slight fluctuations were observed at some concentrations. However, these changes were not biologically meaningful, as cell viability remained within the non-cytotoxic range. No statistically significant differences in cell viability were detected between the untreated control and cells exposed to either the WEGL or EEGL across the entire concentration range tested (one-way ANOVA followed by Tukey’s multiple comparison test, *p* > 0.05). As shown in Figure 4B, WEGPuPe exhibited minimal cytotoxicity toward human chondrocytes across the tested concentration range. Cell viability remained above the generally accepted cytotoxicity threshold of 80%, indicating that the extract was well tolerated by C28/I2 cells under the experimental conditions. The viability of WEGPuPe-treated cells remained relatively stable over the concentration range tested, with only minor fluctuations in mitochondrial metabolic activity. No clear concentration-dependent decrease in cell viability was observed, suggesting that increasing concentrations of WEGPuPe did not adversely affect cell survival. Moreover, several concentrations produced viability values close to or slightly exceeding those of the untreated control, indicating that the extract did not impair cellular metabolic function and may have modestly enhanced mitochondrial activity.

In comparison, WEGS exhibited low cytotoxicity toward C28/I2 cells throughout the tested concentration range (Figure 4C). Cell viability remained above the generally accepted cytotoxicity threshold of 80%, indicating that the extract was well tolerated and did not adversely affect cellular metabolic activity. Across the tested concentrations, WEGS-treated cells maintained relatively stable viability, with no clear concentration-dependent reduction in mitochondrial activity. Although slight variations in viability were observed among concentrations, these changes were minimal and did not indicate biologically relevant cytotoxic effects. For several treatments, cell viability remained similar to or modestly exceeded the level observed in untreated cells, suggesting that WEGS did not impair mitochondrial function and may have supported normal cellular metabolism.

#### 3.3.2. Primary Human Articular Chondrocytes (HAC)

**Figure 5:**
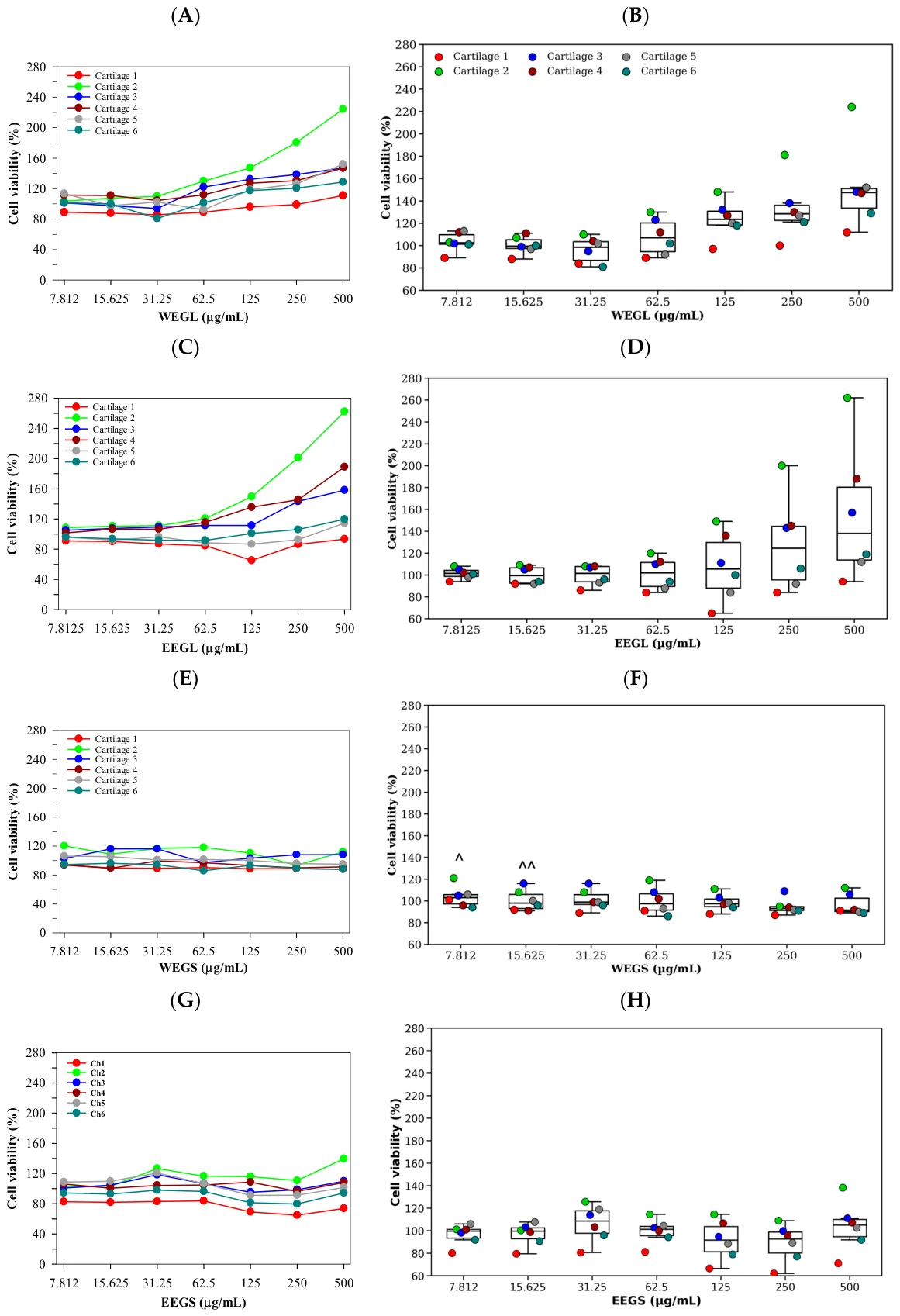

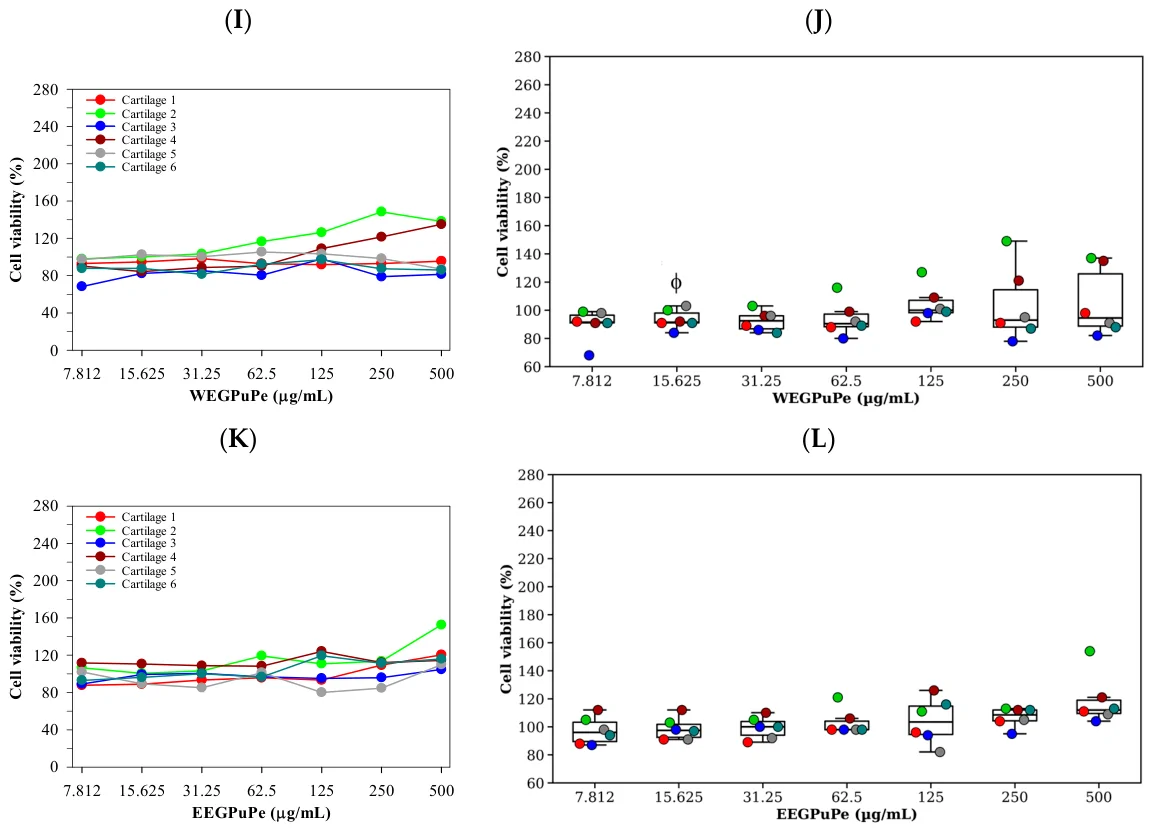
Effects of golden gardenia (*Gardenia sootepensis* Hutch.) extracts on primary human chondrocyte viability after 24 h. (**A**) WEGL dose–response curve, (**B**) WEGL box plot, (**C**) EEGL dose–response curve, (**D**) EEGL box plot, (**E**) WEGS dose–response curve, (**F**) WEGS box plot, (**G**) EEGS dose–response curve, (**H**) EEGS box plot, (**I**) WEGPuPe dose–response curve, (**J**) WEGPuPe box plot, (**K**) EEPuPe dose–response curve, and (**L**) EEPuPe box plot. Results are presented for six independent biological chondrocyte samples (*n* = 6). Differences relative to the untreated control (0 µg/mL) were evaluated by one-way ANOVA followed by Tukey’s multiple-comparison test, with statistical significance denoted as ^^^ *p* < 0.05 and ^^^^ *p* < 0.01 (WEGL vs WEGS); ^ϕ^ *p* < 0.05 (WEGL vs. WEGPuPe).

The effects of water extracts (WE) and ethanolic extracts (EE) of golden gardenia leaves (GL), seeds (GS), and pulp plus peels (GPuPe) on the viability of primary HAC after 24 h of exposure are shown in Figure 5A–L. Overall, cell viability remained above approximately 80% across all tested concentrations, indicating low acute cytotoxicity. Dose-dependent changes were modest and differed among extract types. WEGL and EEGL exhibited minimal effects on chondrocyte viability throughout the tested concentration range, suggesting excellent cytocompatibility (Figure 5A–D). Likewise, WEGS and EEGS produced only slight reductions in viability, with no statistically significant differences between extraction solvents (Figure 5E–H). The pulp-plus-peel extracts (WEGPuPe and EEGPuPe) also demonstrated relatively low cytotoxicity, although the WEGPuPe produced significant reductions in viability at selected concentrations when compared with the untreated control (Figure 5I,K). The corresponding box-and-whisker plots (Figure 5K,L) demonstrate the distribution of cell viability obtained from six independent biological chondrocyte samples. The individual biological values exhibited relatively narrow inter-individual variability across most concentrations, supporting the reproducibility of the observed responses. Most treatment groups overlapped substantially with the untreated control, confirming that the majority of extracts did not induce significant reductions in cell viability after 24 h. Significant differences were observed only in a limited number of high-concentration treatments, particularly for WEGPuPe, whereas EEGL, WEGL, EEGS, and WEGS maintained comparable viability throughout the tested concentration range (Figure 5G–L). These findings indicate that all extracts were generally well tolerated during short-term exposure.

**Figure 6:**
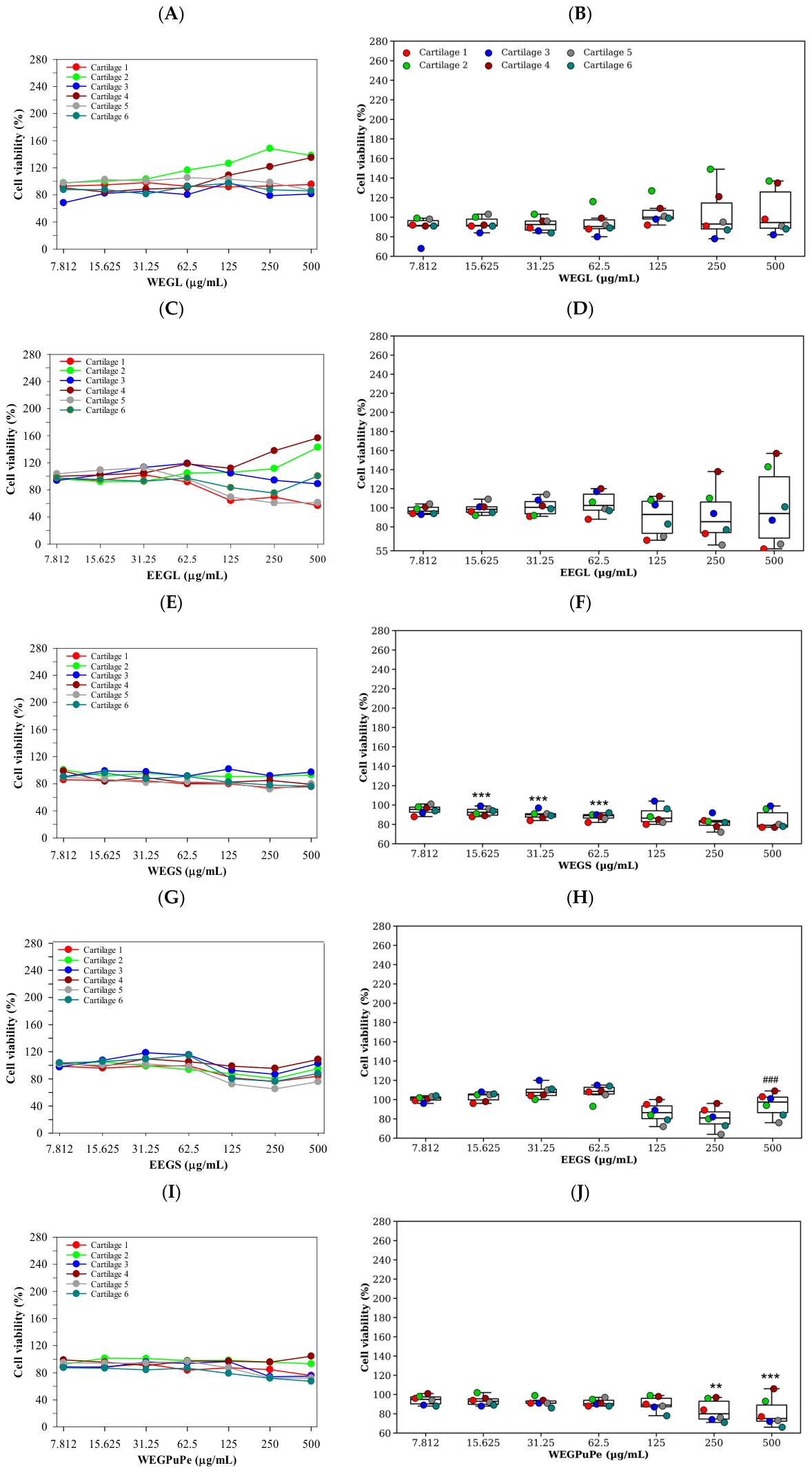

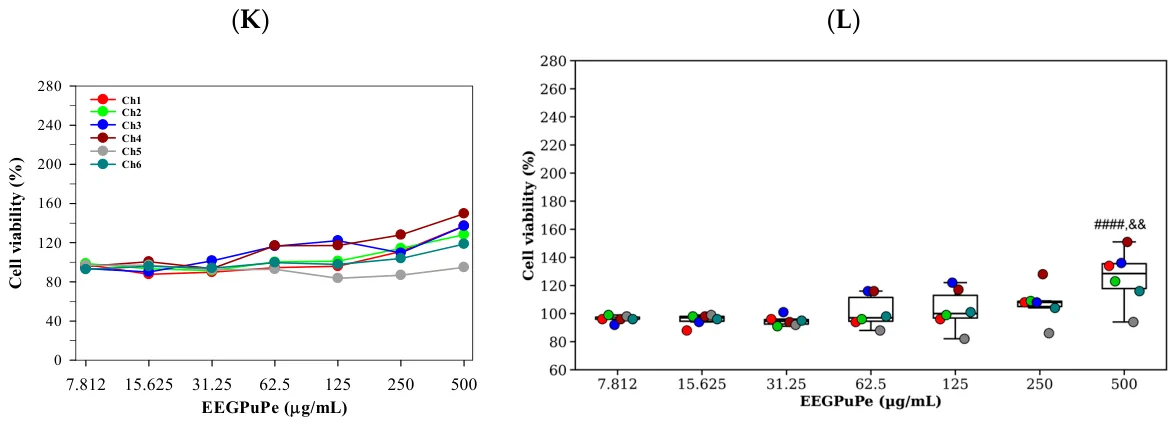
Effects of golden gardenia (*Gardenia sootepensis* Hutch.) extracts on primary human chondrocyte viability after 48 h. (**A**) WEGL dose–response curve, (**B**) EEGL box plot, (**C**) EEGL dose–response curve, (**D**) EEGL box plot, (**E**) WEGS dose–response curve, (**F**) WEGS box plot, (**G**) EEGS dose–response curve, (**H**) EEGS box plot, (**I**) WEGPuPe dose–response curve, (**J**) WEGPuPe box plot, (**K**) EEGPuPe dose–response curve, and (**L**) EEGPuPe box plot. Results represent six independent biological chondrocyte samples (*n* = 6). Comparisons between the untreated control (0 µg/mL) were performed using one-way ANOVA followed by Tukey’s multiple-comparison test, with statistical significance indicated by ** *p* < 0.01 and *** *p* < 0.005 when compared with control (0 μg/mL); ^###^ *p* < 0.01 and ^####^ *p* < 0.005 (EEGL vs EEGS or EEGPuPe); and ^&&^ *p* < 0.01 (EEGS vs EEPuPe).

The effects of prolonged (48 h) exposure of primary human chondrocytes to the six golden gardenia extracts are shown in Figure 6A–L. Compared with the 24 h treatment, concentration-dependent responses became more apparent after 48 h. WEGL and EEGL continued to exhibit minimal cytotoxicity, with no statistically significant difference detected between the two extraction solvents (Figure 6A–D). In contrast, both GS and GPuPe extracts displayed greater time-dependent effects on cell viability. Significant reductions relative to the untreated control were observed in WEGS, EEGS, WEGPuPe, and EEGPuPe at several concentrations, indicating that prolonged exposure enhanced their biological activity (Figure 6E–L). The box-and-whisker plots shown in Figure 6G–L further illustrate the biological variability among the six independent chondrocyte donors. Compared with 24 h exposure, greater dispersion of the data and more pronounced decreases in median cell viability were observed at higher extract concentrations. WEGS exhibited the greatest concentration-dependent decline in cell viability, with statistically significant reductions at several concentrations. Both WEGPuPe and EEGPuPe also demonstrated moderate but significant decreases after prolonged exposure, whereas EEGL and WEGL maintained relatively stable cell viability with limited inter-individual variations. These results indicate that both extract composition and incubation duration contribute to the cytotoxic responses of primary human chondrocytes, with leaf extracts consistently exhibiting the highest cytocompatibility among the tested plant materials (Figure 6G–L).

Table 5 summarizes the statistical comparison between the water and ethanolic extracts tested at equivalent concentrations, as evaluated by two-way ANOVA with subsequent multiple-comparison analysis. No significant differences were detected between water and ethanolic leaf extracts (WEGL vs. EEGL) at either 24 or 48 h, indicating that the extraction solvent did not influence the cytocompatibility of leaf extracts. Likewise, no significant differences were observed between WEGPuPe and EEGPuPe after 24 h. However, after 48 h, the ethanolic and water pulp-plus-peel extracts differed significantly at 250 and 500 µg/mL (*p* = 0.004 and *p* = 0.001, respectively). For seed extracts, no significant effect of extraction solvent was observed at 24 h. In contrast, significant solvent-related differences emerged after 48 h at concentrations of 15.625, 31.25, and 62.5 µg/mL, with *p* values of 0.0043, 0.0019, and 0.0028, respectively. Collectively, these results indicate that the extraction solvent had little effect on leaf extract activity but significantly influenced the biological responses of seed and pulp-plus-peel extracts after prolonged exposure, demonstrating a significant interaction between the extraction solvent and incubation time.

Furthermore, one-way ANOVA followed by Tukey’s multiple-comparison test revealed concentration-dependent differences among the GL, GS, and PuPe extracts when evaluated separately within each extraction solvent (Table 6). At the 24 h time point, only a limited number of statistically significant differences were detected among the three plant-derived extracts, indicating comparable cytocompatibility during short-term exposure. In contrast, after 48 h, significant differences became evident at several concentrations. Within the water extracts, GS generally exhibited lower cell viability than GL, whereas PuPe showed an intermediate response. Similar trends were observed among the ethanolic extracts, with leaf extracts maintaining the highest cell viability across most concentrations. These findings indicate that the plant parts contributed significantly to the biological activity of golden gardenia extracts, particularly following prolonged incubation.

Collectively, these findings revealed that WEGL, EEGL, WEGPuPe, and WEGS possess good in vitro cytocompatibility with C28/I2 human chondrocytes and can be considered non-cytotoxic at the concentrations evaluated. The preservation of high cell viability supports the use of all the extracts in further investigations of their potential biological activities, including antioxidant, anti-inflammatory, and chondroprotective effects.

### 3.4. Antioxidant Activity

**Figure 7:**
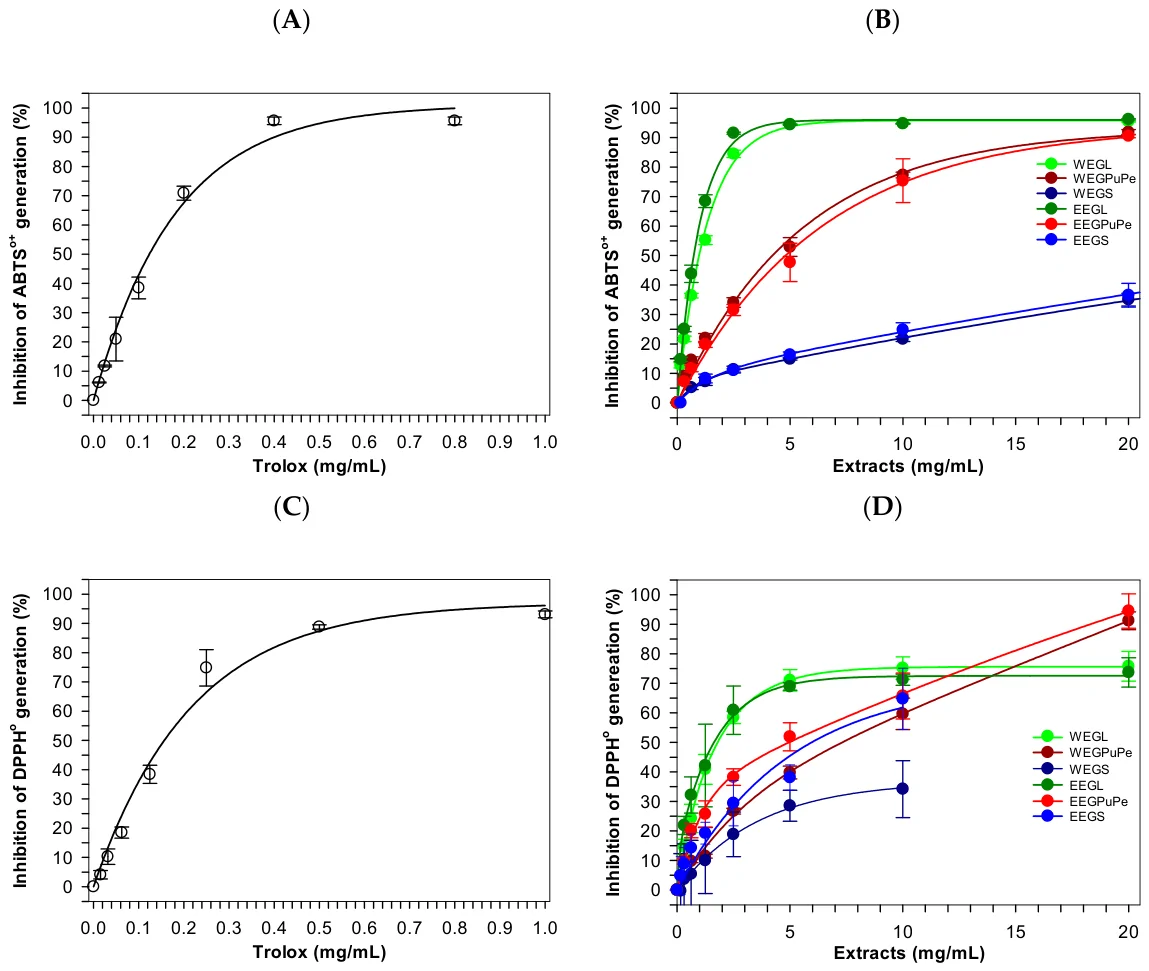
Inhibition of ABTS^•+^ and DPPH^•^ generation by golden gardenia extracts. ABTS^•+^ inhibition by Trolox (**A**) and the extracts (**B**); as well as DPPH^•^ inhibition by Trolox (**C**) and the extracts (**D**). Results are expressed as mean ± SD derived from three independent experiments. Abbreviations: ABTS, 2,2′-azino bis-(3-ethylbenzothiazoline-6-sulfonic acid); DPPH, 2,2-diphenyl-1-picrylhydrazyl; EEGL, ethanolic extract of golden gardenia leaves; EEGS, ethanolic extract of golden gardenia seeds; WEGPuPe, water extract of golden gardenia peel plus pulp; EEGPuPe, ethanolic extract of golden gardenia peel plus pulp; WEGL, water extract of golden gardenia leaves; WEGS, water extract of golden gardenia seeds.

The inhibition of ABTS^•+^ generation increased with increasing extract concentrations in all extracts (Figure 7A), indicating concentration-dependent antioxidant activity. Among the tested extracts, the leaf extracts (WEGL and EEGL) exhibited the strongest ABTS^•+^ radical scavenging activities, achieving nearly complete inhibition (>90%) at concentrations of approximately 2–5 mg/mL. In particular, EEGL showed the highest activity, suggesting that ethanol may have recovered a greater proportion of leaf-derived constituents with ABTS radical-scavenging capacity than water. In contrast, the peel-plus-pulp extracts (WEGPuPe and EEGPuPe) demonstrated moderate activity, reaching approximately 80–90% inhibition only at higher concentrations. The seed extracts (WEGS and EEGS) exhibited the weakest ABTS^•+^ radical scavenging activities, with inhibition values remaining below 40% even at the highest tested concentrations. These findings suggest that antioxidant compounds capable of quenching ABTS radicals were predominantly concentrated in the leaves, followed by the peel plus pulp extract, whereas the seeds contained relatively low levels of these compounds.

A different pattern was observed in the DPPH assay (Figure 7B). The leaf extracts again demonstrated strong radical-scavenging activity, with inhibition values approaching 70–80% at relatively low concentrations. However, the distinction between the leaf and pulp-plus-peel extracts was less marked in the DPPH assay than that observed with the ABTS assay. Both WEGPuPe and EEGPuPe produced considerable DPPH^•^ scavenging activity, suggesting that these extracts contain constituents capable of transferring hydrogen atoms to quench DPPH^•^. Similar to the ABTS results, the seed extracts showed the lowest antioxidant activities. The discrepancy between the ABTS and DPPH assays suggests that different classes of phytochemicals may contribute differently to antioxidant activity depending on the radical system used. ABTS^•+^ can be scavenged by both hydrophilic and lipophilic antioxidants, whereas DPPH^•^ are more selective toward hydrogen-donating compounds. Therefore, the stronger ABTS activity of the leaf extracts may reflect a broader spectrum or higher abundance of phenolic antioxidants present in the leaves.

The AA values of the extracts, together with any significant differences, are shown in Table 7 and varied considerably depending on both plant part and extraction solvent. In the ABTS assay, EEGL exhibited the highest antioxidant capacity (272.92 ± 10.95 mg TE/g), followed by WEGL (235.88 ± 20.66 mg TE/g), with both extracts showing significantly greater activity than the pulp peel and seed extracts (*p* < 0.05). The pulp peel extracts (WEGPuPe and EEGPuPe) displayed moderate antioxidant capacities and were not significantly different from each other, whereas the seed extracts (EEGS and WEGS) exhibited the lowest activities. In contrast, the DPPH assay revealed differences among extracts depending upon the parameters used to express antioxidant activities. WEGPuPe and EEGPuPe showed high Trolox-equivalent antioxidant capacities of 31.25 and 29.32 mg TE/g extract, respectively. However, EEGL and WEGL exhibited the lowest IC_50_ values (1.21 and 2.31 mg/mL, respectively) when compared with WEGPuPe (4.98 mg/mL) and EEGPuPe (7.69 mg/mL), indicating greater DPPH radical-scavenging potency of the leaf extracts on an IC_50_ basis. The leaf extracts demonstrated intermediate DPPH scavenging activities, whereas the seed extracts showed the lowest activities. These findings indicate that antioxidant activity is strongly influenced by both the plant tissue and the radical system employed, suggesting differences in the composition and reactivity of the phytochemical constituents present in the extracts.

The AA of the six golden gardenia extracts were further compared using their IC_50_ values obtained from the ABTS and DPPH radical scavenging assays (Table 7). In the ABTS assay, WEGL and EEGL exhibited the lowest IC_50_ values (0.78 ± 0.06 mg/mL), indicating the strongest radical-scavenging activity. Notably, these values were not significantly different from each other (*p* > 0.05). Both leaf extracts showed significantly greater antioxidant activity than WEGPuPe and EEGPuPe, which displayed intermediate IC_50_ values of 4.64 ± 0.38 and 5.50 ± 1.13 mg/mL, respectively (*p* < 0.05). EEGS demonstrated significantly weaker antioxidant activity with an IC_50_ value of 16.51 ± 1.35 mg/mL (*p* < 0.05), while WEGS failed to achieve 50% ABTS radical inhibition within the tested concentration range, indicating very low antioxidant potency. Similarly, EEGL exhibited the strongest DPPH radical-scavenging activity, along with the lowest IC_50_ value (1.21 ± 0.10 mg/mL), while WEGL showed comparable activity (2.31 ± 0.47 mg/mL) and was not significantly different from EEGL in this regard (*p* > 0.05). EEGPuPe displayed intermediate antioxidant activity (4.98 ± 1.21 mg/mL), whereas WEGPuPe exhibited significantly weaker activity (7.69 ± 0.87 mg/mL) (*p* < 0.05). Nevertheless, IC_50_ values for WEGS in the ABTS assay, and WEGS and EEGS in the DPPH assay, could not be accurately determined because 50% radical inhibition was not achieved within the tested concentration range. Taken together, the leaf extracts (WEGL and EEGL) possessed significantly stronger antioxidant activities than the pulp-peel and seed extracts in both the ABTS and DPPH assays. These results align with the greater total phenolic and flavonoid levels detected in the leaf extracts and suggest that phenolic constituents may be important contributors to the radical-scavenging capacity of *G. sootepensis* extracts

## 4. Discussion

This study presents a comprehendive comparative evaluation of extract yield, phytochemical profile, andtioxidant properties, and chondrocyte cytocompatibility among aqueous and 70% ethanolic extracts derived from different tissues of golden gardenia (*G. sootepensis*). Although several phytochemicals have previously been isolated from golden gardenia flowers and fruits, comparative information regarding leaves, pulp plus peels, and seeds has remained limited [21,22,23,24,42,43]. In the present experiments, seed material generated substantially more dried extract than leaf material, while the leaves—especially after extraction with 70% ethanol—showed greater total phenolic and flavonoid contents. These contrasting patterns indicate that a high extraction yield does not necessarily correspond to enrichment of phenolic constituents. The comparatively large recovery from seeds may instead arise from abundant water- or ethanol-extractable storage compounds, glycosides, or other soluble components. Because proximate composition was not analyzed, the chemical basis for the higher seed yield remains to be established.

Solvent-dependent differences were particularly evident from the TPC and TFC results. Among the six preparations, EEGL showed the highest values for both measurements. Mixtures of ethanol and water can recover a broad spectrum of moderately polar plant metabolites because water facilitates hydration and penetration of plant tissues, whereas ethanol improves solubilization of many phenolic and flavonoid constituents [1,29,30,31]. This complementary solvent behavior explains why aqueous ethanol is frequently selected for the preparation of phytochemical-rich botanical extracts [29,31]. Nevertheless, extraction efficiency should be interpreted on a compound- and tissue-specific basis rather than assuming that ethanol-containing solvents invariably provide greater recovery.

The chromatographic findings reinforce this interpretation. HPLC–ESI–MS revealed chemically distinct profiles containing provisional features attributable to several metabolite classes, particularly iridoid-related compounds, together with phenolic acids, flavonoids, coumarins, and glycosylated phenolics. Iridoid glycosides were among the most prominent groups, with nominal-mass signals tentatively associated with geniposide-, gardenoside-, genipin-, geniposidic acid-, and loganin-related structures occurring in several preparations. The prominence of this chemical class is compatible with previous studies of *Gardenia* species, in which iridoid derivatives constitute characteristic secondary metabolites and have chemotaxonomic relevance [22,23,43,44]. This observation is also pharmacologically relevant because geniposide, genipin, and related compounds have previously been investigated for antioxidant, anti-inflammatory, hepatoprotective, neuroprotective, antifibrotic, and cartilage-associated activities [27,28,29,30,45,46]. These earlier findings provide biological context for the chemical composition observed here but do not demonstrate that the corresponding effects occur in the present extracts. Features provisionally assigned to caffeic acid-, chlorogenic acid-, ferulic acid-, rutin-, quercetin-, and kaempferol-related compounds were also observed. Phenolic acids and flavonoids can participate in antioxidant reactions through electron or hydrogen transfer and stabilization of the resulting radical species [5,16,31].Other reported actions of these compounds include transition-metal binding, attenuation of lipid oxidation, regulation of NF-κB-associated inflammatory signaling, and modulation of Nrf2-dependent antioxidant defenses [5,16]. The occurrence of these metabolite classes, particularly in the ethanolic preparations, is therefore chemically consistent with their antioxidant performance. However, because individual constituents were not quantitatively linked to antioxidant endpoints, the contribution of any specific compound cannot be determined from the current experiments.

An interesting exception to the general solvent pattern was observed for the seed preparations. The low Folin–Ciocalteu value obtained for EEGS should not be interpreted as indicating an absence of phytochemicals. Rather, the result may reflect preferential extraction into water of polar reducing constituents present in seed tissue under the conditions employed. Moreover, the Folin–Ciocalteu reaction is not specific for individual phenolic molecules; it estimates the combined reducing response of phenolics and other substances capable of reacting with the reagent. Consequently, TPC values should be considered together with chromatographic composition and antioxidant measurements rather than used independently as a direct measure of phytochemical abundance. Differences in chromatographic patterns provide further evidence for selective extraction. The water preparations were characterized mainly by earlier-eluting, relatively polar signals, including several iridoid-glycoside-related features. In contrast, extraction with 70% ethanol generated a wider distribution of medium- and later-eluting signals, including features provisionally associated with flavonoids, coumarins, and larger phenolic conjugates. The differences observed between the two solvent systems are compatible with the corresponding TPC and TFC results and illustrate the important influence of solvent polarity on the composition of botanical extracts [22,23,24,25,47,48]. Similar relationships between solvent properties and phytochemical recovery have been reported for other medicinal plants, including *Gardenia jasminoides*, *Mangifera indica*, *Camellia sinensis*, and related antioxidant-rich botanical materials [22,23].

The cellular experiments addressed a different question: whether the extracts were compatible with human chondrocytes at the concentrations examined. Across the six preparations, cell viability generally remained above approximately 80% in both C28/I2 cells and primary human articular chondrocytes. Some pulp-plus-peel and seed preparations produced modest reductions after longer exposure, whereas the leaf preparations generally maintained greater viability. These results support the suitability of the extracts, particularly the leaf preparations, for subsequent experimental investigation. They should, however, be interpreted strictly as evidence of preliminary cytocompatibility rather than as demonstration of cartilage protection. The chemical composition of the leaf preparations provides a rationale for investigating their biological effects further. Polyphenols have previously been associated with reductions in intracellular oxidative stress, preservation of mitochondrial function, regulation of inflammatory mediators, and modulation of enzymes involved in cartilage-matrix degradation [5,9,10,19,20]. Quercetin, kaempferol, and structurally related flavonoids have additionally been reported to influence NF-κB and Nrf2 signaling, responses to interleukin-1β, and extracellular-matrix homeostasis [5,10,19,49,50]. Nevertheless, none of these mechanisms was directly examined in the current work. The present viability data therefore justify mechanistic testing but cannot establish chondroprotective or anti-osteoarthritic activity.

Another consideration concerns the confidence of metabolite identification. The single-quadrupole HPLC–ESI–MS system generated nominal *m*/*z* information in positive-ion mode but did not provide the accurate-mass measurements or diagnostic fragmentation patterns obtainable from high-resolution tandem mass spectrometry. Negative-ion profiles were also unavailable. Accordingly, most assignments should be considered provisional and are appropriately classified mainly within MSI Levels 3–4 rather than as unequivocally identified metabolites. This approach is consistent with MSI principles for communicating confidence in metabolite annotation [29,30,51]. Confirmation will require high-resolution LC–MS/MS using complementary positive- and negative-ion acquisition, preferably with UHPLC–QTOF–MS/MS or Orbitrap-based platforms and comparison with authentic standards. NMR may additionally be necessary for structural verification of selected compounds. Once identities have been established, validated calibration procedures should be applied to determine concentrations of major constituents.

The DPPH findings additionally emphasize the importance of distinguishing between different expressions of antioxidant activity. The relatively high Trolox-equivalent values obtained for the pulp-plus-peel preparations do not mean that these extracts necessarily possessed the greatest radical-scavenging potency. In contrast, the lower IC_50_ values obtained for the leaf preparations indicate that a smaller extract concentration was required to achieve 50% inhibition. Therefore, the two parameters describe different aspects of assay behavior and should be interpreted together. On this basis, the current results do not justify describing the pulp-plus-peel preparations as having the highest overall DPPH antioxidant activity. Taken together, several major observations emerge from the comparison. Plant tissue and extraction solvent independently influenced extract recovery, TPC, TFC, chromatographic complexity, and antioxidant behavior. The seed fractions provided the greatest mass yields, whereas the leaf preparations were enriched in measured phenolic and flavonoid constituents and generally displayed stronger radical-scavenging performance. The HPLC–ESI–MS analysis also indicated that chemical diversity cannot be inferred solely from TPC or TFC because extracts with relatively low colorimetric values may nevertheless contain numerous detectable metabolites. In addition, all preparations exhibited acceptable preliminary compatibility with immortalized and primary human chondrocytes. These complementary findings provide a rational basis for selecting specific extracts for more targeted biological studies. Future studies should determine whether these metabolites protect chondrocytes from oxidative stress, inflammation, ferroptosis, and extracellular matrix degradation in experimental osteoarthritis models and should evaluate their efficacy in vivo before clinical translation [5,9,19,20,44,50,52,53,54]. The study also has several limitations. Most importantly, structural assignments were based on nominal-mass single-quadrupole MS data and therefore remain tentative. Quantitative concentrations of individual constituents, including important markers such as geniposide and chlorogenic acid, were not established using validated analyte-specific methods. Future quantitative work should therefore employ authentic reference compounds and appropriate HPLC-DAD or LC–MS calibration procedures. In addition, ABTS and DPPH measure chemical radical-scavenging reactions under defined in vitro conditions and cannot reproduce intracellular redox regulation. Cellular antioxidant endpoints will consequently be necessary to determine whether the chemical activity measured here translates into biological antioxidant effects. Finally, the chondrocyte experiments assessed viability rather than functional protection against oxidative, inflammatory, or matrix-degrading stimuli. No conclusions concerning therapeutic efficacy can therefore be drawn from the present cellular findings.

Further work should consequently progress from chemical confirmation to mechanism-oriented biological testing. High-resolution UHPLC–QTOF–MS/MS or Orbitrap MS, authentic reference standards, and, when required, NMR should first be used to verify and quantify the principal constituents. Bioactivity-guided fractionation could then determine whether antioxidant effects arise predominantly from individual compounds, particular fractions, or interactions among iridoids, phenolic acids, and flavonoids. In cartilage models, extracts should be tested under defined oxidative and inflammatory challenges, with measurements of intracellular ROS, mitochondrial function, glutathione status, lipid peroxidation, and ferroptosis-related processes. Investigation of Nrf2/HO-1, NF-κB, MAPK, and phosphoinositide-3 kinase/alpha-serine/threonine-protein kinase signaling would help establish whether these pathways contribute to any observed effects [55]. Evaluation of collagen type II, aggrecan, SRY-box transcription factor 9, MMP-1, MMP-3, MMP-13, and ADAMTS-related enzymes would additionally determine whether the extracts influence extracellular-matrix homeostasis under OA-relevant conditions. If reproducible biological activity is subsequently demonstrated, standardized preparations could be advanced to appropriate animal models to characterize exposure, metabolism, safety, dose–response relationships, and efficacy. Such studies are necessary before considering golden gardenia for nutraceutical or phytopharmaceutical applications related to degenerative joint disorders. Future development should therefore be guided by confirmed chemical composition and experimentally demonstrated biological activity rather than by chemical antioxidant assays alone.

Overall, this study provides an integrated comparison of the chemical composition, antioxidant behavior, and preliminary chondrocyte compatibility of water and aqueous-ethanolic preparations from different tissues of *G. sootepensis*. The findings demonstrate that tissue source and solvent selection substantially affect the properties of the resulting extracts. Rather than establishing therapeutic efficacy, the present results identify chemically distinct and cytocompatible preparations that merit further investigation in mechanistic models of oxidative stress, inflammation, and OA-associated cartilage damage.

## 5. Conclusions

Water and 70% ethanolic recovered distinct phytochemical profiles from golden gardenia (*G. sootepensis*) leaves, peel plus pulp, and seeds. Seed preparations produced the highest yields and greatest detectable chemical diversity, whereas leaf extracts contained the highest phenolic and flavonoid contents and showed the strongest radical-scavenging activity. HPLC–ESI–MS features were provisionally assigned to iridoid-related compound, phenolic acids, flavonoids and their glycosides, xanthones, and other specialized metabolites. All preparations maintained favorable viability in immortalized and primary human chondrocytes under the conditions tested. These findings support further investigation of standardized *G. sootepensis* extracts, while high-resolution structural confirmation and disease-relevant in vitro and in vivo studies remain necessary before efficacy or cartilage-protective claims can be made.

## Figures and Tables

**Table 1:** Clinical characteristics of donors providing cartilage specimens.

Specimen Code	Gender	Age (y)	Diagnosis	Treatment
Cartilage 1	Male	68	Primary osteoarthritis	Pharmacological treatment and physical therapy
Cartilage 2	Female	68	Primary osteoarthritis	Pharmacological treatment and physical therapy
Cartilage 3	Female	68	Primary osteoarthritis	Pharmacological treatment and physical therapy
Cartilage 4	Female	59	Primary osteoarthritis	Pharmacological treatment and physical therapy
Cartilage 5	Female	45	Primary osteoarthritis	Pharmacological treatment and physical therapy
Cartilage 6	Female	53	Primary osteoarthritis	Pharmacological treatment and physical therapy

**Table 2:** Fresh weight, dry weight, extract weight, and extraction yields of water and ethanolic extracts prepared from leaves, peels plus pulp, and seeds of golden gardenia (*Gardenia sootepensis* Hutch.).

Sample	Fresh	Dry	Extract	Dry	Extraction Yield	
	Weight (g)	Weight (g)		Weight (g)	(mg/g Dry Weight)	(mg/g Fresh Weight)
Leaves	1600	250	WEGL	2.66	10.62	1.06
Leaves	1600	250	EEGL	2.81	11.23	1.12
Peel-Pulp	500	340	WEGPuPe	20.36	59.89	5.99
Peel-Pulp	500	320	EEGPuPe	9.16	28.61	2.86
Seeds	500	100	WEGS	11.62	116.17	11.62
Seeds	500	140	EEGS	10.70	76.41	7.64

Abbreviations: EEGL, ethanolic extract of golden gardenia leaves; EEGPuPue ethanolic extract of golden gardenia peel plus pulp; EEGS, ethanolic extract of golden gardenia seeds; WEGL, water extract of golden gardenia leaves; WEGPuPe, water extract of golden gardenia peel plus pulp; WEGS, water extract of golden gardenia seeds.

**Table 3:** Total phenolic content (TPC) and total flavonoid content (TFC) of water and ethanolic extracts prepared from different parts of golden gardenia (*Gardenia sootepensis* Hutch.). Data obtained from three separate determinations are presented as mean ± SD values.

Sample	TPC (mg GAE/g)	TFC (mg QE/g)
WEGL	71.72 ± 4.06 ^b^	24.15 ± 1.91 ^a^
EEGL	81.08 ± 4.60 ^a^	28.36 ± 3.63 ^a^
WEGPuPe	25.01 ± 0.45 ^d^	2.06 ± 0.15 ^c^
EEGPuPe	33.11 ± 0.70 ^c^	7.37 ± 0.34 ^b^
WEGS	19.12 ± 0.12 ^e^	24.38 ± 0.08 ^c^
EEGS	0.43 ± 0.30 ^e^	2.47 ± 0.47 ^c^

Different superscript letters within the same column indicate statistically significant differences among extracts (one-way ANOVA followed by Tukey’s HSD multiple-comparison test (*p* < 0.05)). Abbreviations: EEGL, ethanolic extract of golden gardenia leaves; EEGPuPe, ethanolic extract of golden gardenia peel plus pulp; EEGS, ethanolic extract of golden gardenia seeds; GAE, gallic acid equivalent; QE, quercetin equivalent; TFC, total flavonoid content; TPC, total phenolic content; WEGL, water extract of golden gardenia leaves; WEGPuPe, water extract of golden gardenia peel plus pulp; WEGS, water extract of golden gardenia seeds.

**Table 4:** Provisional annotation of phytochemical features detected across six golden gardenia (*Gardenia sootepensis* Hutch.) by high-performance liquid chromatography-electrospray ionization-mass spectrometry (HPLC-ESI-MS).

Peak	T_R_ (min)	Experimental *m*/*z*	PMF	Tentative Compound	Chemical Class	MSI Level	WEGL	EEGL	WEGPuPe	EEGPuPe	WEGS	EEGS
Iridoids glycosides and derivatives												
1	2.28	127.0	C_6_H_6_O_3_	Hydroxybenzoic acid derivative	Iridoid precursor	4	✓	✓	✓	✓	✓	✓
2	2.52	210.1	C_7_H_10_O_7_	Gluconic acid	Iridoid precursor	4	✓	✓	–	–	✓	✓
3	2.82	209.0	C_7_H_9_O_7_	Dehydroascorbic acid	Iridoid precursor	4	✓	✓	–	–	✓	✓
4	3.04	223.1	C_8_H_10_O_7_	Loganin (aglycone)	Iridoid	3	✓	✓	✓	✓	✓	✓
5	3.39	241.1	C_10_H_12_O_7_	Secoxyloganin (aglycone)	Iridoid	3	✓	✓	✓	✓	✓	✓
6	9.82	399.1	C_17_H_22_O_11_	Geniposide	Iridoid glycoside	2	✓	✓	✓	✓	✓	✓
7	10.11	399.1	C_17_H_22_O_11_	Gardenoside	Iridoid glycoside	2	✓	✓	✓	✓	✓	✓
8	10.48	227.1	C_10_H_10_O_6_	Genipin (aglycone)	Iridoid	2	✓	✓	✓	✓	✓	✓
9	11.32	447.1	C_21_H_22_O_11_	Gardenoside methyl ester	Iridoid glycoside	2	✓	✓	✓	✓	✓	✓
10	11.7	229.1	C_10_H_12_O_6_	7-Deoxyloganin (aglycone)	Iridoid	3	✓	✓	✓	✓	✓	✓
11	11.73	225.1	C_10_H_10_O_6_	7-Epideoxyloganin (aglycone)	Iridoid	3	✓	✓	✓	✓	✓	✓
12	12.06	223.1	C_10_H_10_O_6_	10-Hydroxygeniposide(aglycone)	Iridoid	3	✓	✓	✓	✓	✓	✓
13	12.57	225.1	C_10_H_10_O_6_	Geniposidic acid (aglycone)	Iridoid	3	✓	✓	✓	✓	✓	✓
14	13.2	209.0	C_8_H_8_O_6_	Shanzhiside methyl ester	Iridoid	3	✓	✓	✓	✓	✓	✓
15	13.46	227.1	C_10_H_10_O_6_	Geniposidic acid methyl ester	Iridoid	3	✓	✓	✓	✓	✓	✓
Phenolic acids and derivatives												
16	13.59	191.1	C_8_H_10_O_5_	Quinic acid	Phenolic acid	2	✓	✓	✓	✓	✓	✓
17	15.04	235.1	C_12_H_10_O_5_	Chlorogenic acid	Phenolic acid	2	✓	✓	✓	✓	✓	✓
18	17.33	193.1	C_9_H_8_O_4_	Caffeic acid	Phenolic acid	2	✓	✓	✓	✓	✓	✓
19	18.11	310.1	C_15_H_14_O_7_	Rutin (quercetin-3-O-rutinoside)	Phenolic glycoside	2	✓	✓	✓	✓	✓	✓
20	21.97	179.0	C_5_H_6_O_4_	Ferulic acid	Phenolic acid	2	✓	✓	✓	✓	✓	✓
Flavonoids and glycosides												
21	24.02	463.1	C_21_H_20_O_12_	Quercetin-3-O-glucoside	Flavonoid glycoside	2	✓	✓	✓	✓	✓	✓
22	24.57	481.1	C_22_H_22_O_12_	Quercetin-3-O-rutinoside	Flavonoid glycoside	2	✓	✓	✓	✓	✓	✓
23	25.31	449.1	C_21_H_20_O_11_	Kaempferol-3-O-glucoside	Flavonoid glycoside	2	✓	✓	✓	✓	✓	✓
24	25.76	433.1	C_20_H_20_O_11_	Kaempferol-3-O-rutinoside	Flavonoid glycoside	2	✓	✓	✓	✓	✓	✓
25	26.58	303.1	C_15_H_10_O_7_	Quercetin	Flavonoid	2	✓	✓	✓	✓	✓	✓
26	27.49	287.1	C_15_H_10_O_6_	Kaempferol	Flavonoid	2	✓	✓	✓	✓	✓	✓
27	28.36	317.1	C_16_H_12_O_7_	Isorhamnetin	Flavonoid	2	✓	✓	✓	✓	✓	✓
28	29.25	595.1	C_27_H_30_O_15_	Quercetin-3-rutinoside-pentoside	Flavonoid glycoside	3	✓	✓	✓	✓	✓	✓
29	30.12	609.1	C_28_H_32_O_15_	Acetylated hyperosides	Flavonoid glycoside	3	–	✓	–	✓	–	✓
Other phenolics/miscellaneous												
30	31.87	301.1	C_16_H_12_O_6_	Xanthone derivative	Xanthone	3	✓	✓	✓	✓	✓	✓
31	34.08	343.1	C_18_H_14_O_7_	Xanthone glycoside	Xanthone glycoside	3	✓	✓	✓	✓	✓	✓
32	37.26	265.1	C_15_H_12_O_4_	Benzophenone derivative	Benzophenone glycoside	3	✓	✓	✓	✓	✓	✓
33	44.72	515.1	C_25_H_26_O_12_	Iridoid–phenolic conjugate	Iridoid–conjugate	3	–	✓	–	✓	–	✓
34	55.83	676.2	C_33_H_40_O_15_	HMW polyphenolic conjugate	Polyphenol conjugate	4	–	✓	–	✓	–	✓

Note: Identifications and molecular formulas are provisional and should be verified against original instrument data, accurate-mass HPLC–MS/MS spectra, isotope patterns, and authentic standards. Abbreviations/Symbols: WEGL, water extract of golden gardenia leaves; EEGL, ethanolic extract of golden gardenia leaves; WEGPuPe, water extract of golden gardenia pulp plus peel; EEGPuPe, ethanolic extract of golden gardenia pulp plus peel; WEGS, water extract of golden gardenia seeds; EEGS, ethanolic extract of golden gardenia seeds; HMW, high molecular weight; MSI, Metabolomics Standards Initiative; PMF, proposed molecular formula; T_R_, retention time; ✓, detectable; –, undetectable.

**Table 5:** Two-way ANOVA followed by Tukey’s multiple—comparison analyses comparing water (WE) and ethanolic (EE) extracts of *G. sootepensis* Hutchs leaves (GL), pulp plus peel (GPuPe), and seeds (GS) at equivalent concentrations after 24 and 48 h of treatment in primary human chondrocytes (*n* = 6 biological samples).

Extract Type	Incubation	Concentration (μg/mL)	*p* Value
WEGL	24 h	All concentrations	>0.05
WEGL	48 h	All concentrations	>0.05
WEGPuPe	24 h	All concentrations	>0.05
WEGPuPe	48 h	250	<0.01
		500	<0.005
WEGS	24 h	All concentrations	>0.05
	48 h	15.625	<0.005
		31.25	<0.005
		62.5	<0.005
		125	>0.05
		250	>0.05
		500	>0.05

Data are presented as mean ± SD values from six independent biological chondrocyte samples (*n* = 6). Statistical analyses were performed using two-way ANOVA with extraction solvent and concentration as independent factors, followed by Tukey’s multiple-comparison test to compare WE and EE at each equivalent concentration.

**Table 6:** Statistical comparison among leaves (GL), pulp plus peel (PuPe), and seeds (GS) extracts at corresponding concentrations with each extraction solvent after 24 and 48 h of exposure in primary human chondrocytes (*n* = 6 independent biological samples), as evaluated by one-way ANOVA and Tukey’s multiple-comparison test.

Solvent	Time	Concentration	*p* Value		
		(μg/mL)	GL vs. GS	GL vs. PuPe	GS vs. PuPe
WE	24 h	0	ns	ns	ns
WE	24 h	7.812	<0.05	ns	ns
WE	24 h	15.625	<0.01	<0.05	ns
EE	48 h	500	<0.005	<0.001	<0.01

**Table 7:** Antioxidant activity (AA) and IC_50_ values of water and ethanolic extracts prepared from different parts of golden gardenia (*Gardenia sootepensis* Hutch.) determined by ABTS and DPPH assays. Results from three independent experiments are presented as the mean ± SD and reported as milligrams of Trolox equivalents per gram of extract (mg TE/g extract). The IC_50_ denotes the extract concentration required to achieve 50% scavenging of ABTS or DPPH radicals; consequently, a lower IC_50_ corresponds to stronger antioxidant activity.

Sample	ABTS Assay		DPPH Assay	
	AA (mg TE/g)	IC_50_ (mg/mL)	AA (mg TE/g)	IC_50_ (mg/mL)
Trolox	-	0.13 ± 0.02	-	0.17 ± 0.01
WEGL	235.88 ± 20.66 ^b^	0.78 ± 0.26 ^a^	12.17 ± 0.29 ^c^	2.31 ± 0.47 ^ab^
EEGL	272.92 ± 10.95 ^a^	0.78 ± 0.06 ^a^	11.72 ± 0.26 ^c^	1.21 ± 0.10 ^a^
WEGPuPe	31.25 ± 1.16 ^c^	4.64 ± 0.38 ^b^	31.25 ± 1.16 ^a^	7.69 ± 0.87 ^c^
EEGPuPe	29.32 ± 2.62 ^c^	5.50 ± 1.13 ^b^	29.32 ± 2.62 ^a^	4.98 ± 1.21 ^b^
WEGS	5.81 ± 0.20 ^d^	>40	2.55 ± 0.37 ^d^	>10
EEGS	10.65 ± 0.64 ^d^	16.51 ± 1.35 ^c^	4.83 ± 0.50 ^b^	>10

Within each column, different superscript letters denote significant differences among the extracts, as determined by one-way ANOVA followed by Tukey’s multiple comparison test (*p* < 0.05). Abbreviations: ABTS, 2,2′-azino bis-(3-ethylbenzothiazoline-6-sulfonic acid); DPPH, 2,2-diphenyl-1-picrylhydrazyl; EEGL, ethanolic extract of golden gardenia leaves; EEGPuPe, ethanolic extract of golden gardenia peel plus pulp; IC_50_, half-maximal inhibitory concentration; TE, Trolox equivalents; WEGL, water extract of golden gardenia leaves; WEGPuPe, water extract of golden gardenia peel plus pulp; WEGS, water extract of golden gardenia seeds; EEGS, ethanolic extract of golden gardenia seeds.

## Data Availability

The original contributions presented in this study are included in the article/Supplementary Materials. Further inquiries can be directed to the corresponding authors.

## Supplementary Materials

The following supporting information can be downloaded at: https://www.mdpi.com/article/10.3390/antiox15091083/s1, Table S1. Provisional annotation of high-performance liquid chromatography/electrospray ionization-mass spectrometry (HPLC-ESI-MS) (positive-ion mode) features detected in the water extract of Golden Gardenia (*Gardenia sootepensis*) leaves (WEGL). Table S2. Provisional annotation of HPLC-ESI-MS (positive-ion mode) features detected in the ethanolic extract of Golden Gardenia (*G. sootepensis*) leaves (EEGL). Table S3. Provisional annotation of HPLC-ESI-MS (positive-ion mode) features detected in the water extract of Golden Gardenia (*G. sootepensis*) pulp plus peels (WEGPuPe); Table S4. Provisional annotation of HPLC-ESI-MS (positive-ion mode) features detected in the ethanolic extract of Golden Gardenia (*G. sootepensis*) pulp plus peels (EEGPuPe); Table S5. Provisional annotation of HPLC-ESI-MS (positive-ion mode) features detected in the water extract of Golden Gardenia (*G. sootepensis*) seeds (WEGS); and Table S6. Provisional annotation of HPLC-ESI-MS (positive-ion mode) features detected in the ethanolic extract of Golden Gardenia (*G. sootepensis*) seeds (EEGS). Table S7. Comparative distribution of provisionally annotated HPLC-ESI-MS (positive-ion mode) phytochemical features across WEGL, EEGL, WEGPuPe, EEGPuPe, WEGS, and EEGS.

## Abbreviations

The following abbreviations are used in this manuscript:

A	Acetonitrile
AA	Antioxidant activity
ABTS	2,2′-Azino-bis(3-ethylbenzothiazoline-6-sulfonic acid
ANOVA	Analysis of variance
B	10 mM Formate buffer pH 4.0
CDU	Collagen-digesting unit
DI	Deionized water
DMEM	Dulbecco’s modified Eagle’s medium
DMSO	Dimethyl sulfoxide
DPPH	2,2-Diphenyl-1-picrylhydrazyl
EEGL	Ethanolic extract of golden gardenia leaves
EEGPePu	Ethanolic extract of golden gardenia peel + pulp
EEGS	Ethanolic extract of golden gardenia seeds
Expt	Experiment
FBS	Fetal bovine serum
*G. sootepensis*	*Gardenia sootepensis*
GA	Gallic acid
GAE	Gallic acid equivalent
H	High
HAC	Human articular chondrocytes
HO-1	Heme oxygenase-1
HPLC–ESI–MS	High-performance liquid chromatography coupled with electrospray ionization mass spectrometry
ICH-GCP	International Council for Harmonisation Guideline for Good Clinical Practice
IC_50_	Half-maximal inhibitory concentration
L	Low
M	Medium
MAPK	Mitogen-activated protein kinase
MMP	Matrix metalloproteinases
MSI	Metabolomics Standards Initiative
MTT	[3-(4,5-Dimethylthiazol-2-yl)-2,5-diphenyltetrazolium bromide]
*m*/*z*	Mass-to-charge ratio
NF-κB	Nuclear factor-kappa B
NMR	Nuclear magnetic resonance
Nrf2	Nuclear factor erythroid 2-related factor 2
OA	Osteoarthritis
PBS	Phosphate-buffered saline
PDA	Photodiode array
PMF	Proposed molecular formula
Q	Quercetin
QE	Quercetin equivalent
ROS	Reactive oxygen species
SD	Standard deviation
SEM	Standard error of the mean
TFC	Total flavonoid content
TPC	Total phenolic content
Trolox	6-Hydroxy-2,5,7,8-tetramethylchromane-2-carboxylic
UHPLC–QTOF–MS/MS	Ultrahigh-pressure liquid chromatography-quadrupole-time-of flight tandem mass spectrometry
UV/VIS	Ultraviolet/visible
*v*/*v*	Volume by volume
*w*/*v*	Weight by volume
WEGL	Water extracts of golden gardenia leaves
WEGPePu	Water extracts of golden gardenia peel + pulp
WEGS	Water extracts of golden gardenia seeds
