# Supplementary material for: Comparative Phytochemical Profiling, Antioxidant Activity, and Chondrocyte Cytocompatibility of Water and Ethanolic Extracts from Different Organs of Golden Gardenia (Gardinia sootepensis)
Source: Antioxidants (Basel). 2026 Aug 28;15(9):1083. doi: 10.3390/antiox15091083 (PMC13603909; doi:10.3390/antiox15091083)
Supplement: Supplementary file 1 [file antioxidants-15-01083-s001.zip › antioxidants-4473137-supplementary.pdf]

Supplementary Information **Table S1.** Provisional annotation of high-performance liquid chromatography/electrospray ionization-mass spectrometry (HPLC-ESI-MS) (positive-ion mode) features detected in the water extract of Golden Gardenia (*Gardenia sootepensis*) leaves (WEGL).

Peak No.	T_R (min)[1]	Experimental m/z	Proposed molecular formula[2]	Tentative compound	Compound class	MSI level[3]
1	2.28	127.0	$C_6H_6O_3$	Hydroxybenzoic acid derivative	Iridoid precursor	4
2	2.52	210.1	$C_7H_{10}O_7$	Gluconic acid	Iridoid precursor	4
3	2.82	209	$C_7H_9O_7$	Dehydroascorbic acid	Iridoid precursor	4
4	3.04	223.1	$C_8H_{10}O_7$	Loganin (aglycone)	Iridoid	3
5	3.39	241.1	$C_{10}H_{12}O_7$	Secoxyloganin (aglycone)	Iridoid	3
6	9.82	399.1	$C_{17}H_{22}O_{11}$	Geniposide	Iridoid glycoside	2
7	10.11	399.1	$C_{17}H_{22}O_{11}$	Gardenoside	Iridoid glycoside	2
8	10.48	227.1	$C_{10}H_{10}O_6$	Genipin (aglycone)	Iridoid	2
9	11.32	447.1	$C_{21}H_{22}O_{11}$	Gardenoside methyl ester	Iridoid glycoside	2
10	11.70	229.1	$C_{10}H_{12}O_6$	7-Deoxyloganin (aglycone)	Iridoid	3
11	11.73	225.1	$C_{10}H_{10}O_6$	7-Epideoxyloganin (aglycone)	Iridoid	3
12	12.06	223.1	$C_{10}H_{10}O_6$	10-Hydroxygeniposide (aglycone)	Iridoid	3
13	12.57	225.1	$C_{10}H_{10}O_6$	Geniposidic acid (aglycone)	Iridoid	3
14	13.20	209.0	$C_8H_8O_6$	Shanzhiside methyl ester	Iridoid	3
15	13.46	227.1	$C_{10}H_{10}O_6$	Geniposidic acid methyl ester	Iridoid	3
16	13.59	191.1	$C_8H_{10}O_5$	Quinic acid	Phenolic acid	2
17	15.04	235.1	$C_{12}H_{10}O_5$	Chlorogenic acid	Phenolic acid	2
18	17.33	193.1	$C_9H_8O_4$	Caffeic acid	Phenolic acid	2
19	18.11	310.1	$C_{15}H_{14}O_7$	Rutin (quercetin-3-O-rutinoside)	Phenolic glycoside	2
20	21.97	179.0	$C_5H_6O_4$	Ferulic acid	Phenolic acid	2
21	24.02	463.1	$C_{21}H_{20}O_{12}$	Quercetin-3-O-glucoside	Flavonoid glycoside	2
22	24.57	481.1	$C_{22}H_{22}O_{12}$	Quercetin-3-O-rutinoside	Flavonoid glycoside	2
23	25.31	449.1	$C_{21}H_{20}O_{11}$	Kaempferol-3-O-glucoside	Flavonoid glycoside	2
24	25.76	433.1	$C_{20}H_{20}O_{11}$	Kaempferol-3-O-rutinoside	Flavonoid glycoside	2
25	26.58	303.1	$C_{15}H_{10}O_7$	Quercetin	Flavonoid	2
26	27.49	287.1	$C_{15}H_{10}O_6$	Kaempferol	Flavonoid	2
27	28.36	317.1	$C_{16}H_{12}O_7$	Isorhamnetin	Flavonoid	2
28	29.25	595.1	$C_{27}H_{30}O_{15}$	Quercetin-3-rutinoside-pentoside	Flavonoid glycoside	3
30	31.87	301.1	$C_{16}H_{12}O_6$	Xanthone derivative	Xanthone	3

Peak No.	T_R (min)[1]	Experimental m/z	Proposed molecular formula[2]	Tentative compound	Compound class	MSI level[3]
31	34.08	343.1	$C_{18}H_{14}O_7$	Xanthone glycoside	Xanthone glycoside	3
32	37.26	265.1	$C_{15}H_{12}O_4$	Benzophenone derivative	Benzophenone glycoside	3

[1] Retention times were read from the handwritten annotations accompanying the individual spectral printouts and should be verified against the original instrument data file.

[2] Proposed molecular formula is provided only when a plausible precursor/adduct assignment can be made. "Undetermined" indicates that the nominal-mass MS spectrum does not support a unique elemental formula. It is proposed only where the nominal mass and known Gardenia chemistry provide a reasonable candidate. They were not calculated from high-resolution accurate masses.

[3]MSI 3 indicates a putatively characterized compound class or tentative structure based on mass and published occurrence; MSI 4 indicates an unknown feature or tentative chemical class. None of the assignments reached MSI 1 or 2 because authentic standards and diagnostic MS-MS spectra were not provided.

Abbreviations: T_R, retention time; m/z, mass-to-charge ratio; MSI, Metabolomics Standards Initiative. Compound names, molecular formulas, and MSI levels are reproduced from the source manuscript annotation and are provisional. They should be verified against the original instrument data, accurate-mass HPLC-MS/MS spectra, isotope patterns, and authentic standards before publication.

Table S2. Provisional annotation of HPLC-ESI-MS (positive-ion mode) features detected in the ethanolic extract of Golden Gardenia (*G. sootepensis*) leaves (EEGL).

Peak No.	T_R (min)[1]	Experimental m/z	Proposed molecular formula[2]	Tentative compound	Compound class	MSI level[3]
1	2.28	127	$C_6H_6O_3$	Hydroxybenzoic acid derivative	Iridoid precursor	4
2	2.52	210.1	$C_7H_{10}O_7$	Gluconic acid	Iridoid precursor	4
3	2.82	209	$C_7H_9O_7$	Dehydroascorbic acid	Iridoid precursor	4
4	3.04	223.1	$C_8H_{10}O_7$	Loganin (aglycone)	Iridoid	3
5	3.39	241.1	$C_{10}H_{12}O_7$	Secoxyloganin (aglycone)	Iridoid	3
6	9.82	399.1	$C_{17}H_{22}O_{11}$	Geniposide	Iridoid glycoside	2
7	10.11	399.1	$C_{17}H_{22}O_{11}$	Gardenoside	Iridoid glycoside	2
8	10.48	227.1	$C_{10}H_{10}O_6$	Genipin (aglycone)	Iridoid	2
9	11.32	447.1	$C_{21}H_{22}O_{11}$	Gardenoside methyl ester	Iridoid glycoside	2
10	11.70	229.1	$C_{10}H_{12}O_6$	7-Deoxyloganin (aglycone)	Iridoid	3
11	11.73	225.1	$C_{10}H_{10}O_6$	7-Epideoxyloganin (aglycone)	Iridoid	3
12	12.06	223.1	$C_{10}H_{10}O_6$	10-Hydroxygeniposide (aglycone)	Iridoid	3
13	12.57	225.1	$C_{10}H_{10}O_6$	Geniposidic acid (aglycone)	Iridoid	3
14	13.20	209	$C_8H_8O_6$	Shanzhiside methyl ester	Iridoid	3
15	13.46	227.1	$C_{10}H_{10}O_6$	Geniposidic acid methyl ester	Iridoid	3
16	13.59	191.1	$C_8H_{10}O_5$	Quinic acid	Phenolic acid	2
17	15.04	235.1	$C_{12}H_{10}O_5$	Chlorogenic acid	Phenolic acid	2
18	17.33	193.1	$C_9H_8O_4$	Caffeic acid	Phenolic acid	2
19	18.11	310.1	$C_{15}H_{14}O_7$	Rutin (quercetin-3-O-rutinoside)	Phenolic glycoside	2
20	21.97	179	$C_5H_6O_4$	Ferulic acid	Phenolic acid	2
21	24.02	463.1	$C_{21}H_{20}O_{12}$	Quercetin-3-O-glucoside	Flavonoid glycoside	2
22	24.57	481.1	$C_{22}H_{22}O_{12}$	Quercetin-3-O-rutinoside	Flavonoid glycoside	2
23	25.31	449.1	$C_{21}H_{20}O_{11}$	Kaempferol-3-O-glucoside	Flavonoid glycoside	2
24	25.76	433.1	$C_{20}H_{20}O_{11}$	Kaempferol-3-O-rutinoside	Flavonoid glycoside	2
25	26.58	303.1	$C_{15}H_{10}O_7$	Quercetin	Flavonoid	2
26	27.49	287.1	$C_{15}H_{10}O_6$	Kaempferol	Flavonoid	2
27	28.36	317.1	$C_{16}H_{12}O_7$	Isorhamnetin	Flavonoid	2
28	29.25	595.1	$C_{27}H_{30}O_{15}$	Quercetin-3-rutinoside-pentoside	Flavonoid glycoside	3
29	30.12	609.1	$C_{28}H_{32}O_{15}$	Acetylated hyperosides	Flavonoid glycoside	3

Peak No.	T_R (min)[1]	Experimental m/z	Proposed molecular formula[2]	Tentative compound	Compound class	MSI level[3]
30	31.87	301.1	$C_{16}H_{12}O_6$	Xanthone derivative	Xanthone	3
31	34.08	343.1	$C_{18}H_{14}O_7$	Xanthone glycoside	Xanthone glycoside	3
32	37.26	265.1	$C_{15}H_{12}O_4$	Benzophenone derivative	Benzophenone glycoside	3
33	44.72	515.1	$C_{25}H_{26}O_{12}$	Iridoid–phenolic conjugate	Iridoid–conjugate	3
34	55.83	676.2	$C_{33}H_{40}O_{15}$	HMW polyphenolic conjugate	Polyphenol conjugate	4

Table S3. Provisional annotation of HPLC-ESI-MS (positive-ion mode) features detected in the water extract of Golden Gardenia (*G. sootepensis*) pulp plus peel (WEGPuPe).

Peak No.	T_R (min)[1]	Experimental m/z	Proposed molecular formula[2]	Tentative compound	Compound class	MSI level[3]
1	2.28	127	$C_6H_6O_3$	Hydroxybenzoic acid derivative	Iridoid precursor	4
4	3.04	223.1	$C_8H_{10}O_7$	Loganin (aglycone)	Iridoid	3
5	3.39	241.1	$C_{10}H_{12}O_7$	Secoxyloganin (aglycone)	Iridoid	3
6	9.82	399.1	$C_{17}H_{22}O_{11}$	Geniposide	Iridoid glycoside	2
7	10.11	399.1	$C_{17}H_{22}O_{11}$	Gardenoside	Iridoid glycoside	2
8	10.48	227.1	$C_{10}H_{10}O_6$	Genipin (aglycone)	Iridoid	2
9	11.32	447.1	$C_{21}H_{22}O_{11}$	Gardenoside methyl ester	Iridoid glycoside	2
10	11.70	229.1	$C_{10}H_{12}O_6$	7-Deoxyloganin (aglycone)	Iridoid	3
11	11.73	225.1	$C_{10}H_{10}O_6$	7-Epideoxyloganin (aglycone)	Iridoid	3
12	12.06	223.1	$C_{10}H_{10}O_6$	10-Hydroxygeniposide (aglycone)	Iridoid	3
13	12.57	225.1	$C_{10}H_{10}O_6$	Geniposidic acid (aglycone)	Iridoid	3
14	13.20	209	$C_8H_8O_6$	Shanzhiside methyl ester	Iridoid	3
15	13.46	227.1	$C_{10}H_{10}O_6$	Geniposidic acid methyl ester	Iridoid	3
16	13.59	191.1	$C_8H_{10}O_5$	Quinic acid	Phenolic acid	2
17	15.04	235.1	$C_{12}H_{10}O_5$	Chlorogenic acid	Phenolic acid	2
18	17.33	193.1	$C_9H_8O_4$	Caffeic acid	Phenolic acid	2
19	18.11	310.1	$C_{15}H_{14}O_7$	Rutin (quercetin-3-O-rutinoside)	Phenolic glycoside	2
20	21.97	179	$C_5H_6O_4$	Ferulic acid	Phenolic acid	2
21	24.02	463.1	$C_{21}H_{20}O_{12}$	Quercetin-3-O-glucoside	Flavonoid glycoside	2
22	24.57	481.1	$C_{22}H_{22}O_{12}$	Quercetin-3-O-rutinoside	Flavonoid glycoside	2
23	25.31	449.1	$C_{21}H_{20}O_{11}$	Kaempferol-3-O-glucoside	Flavonoid glycoside	2
24	25.76	433.1	$C_{20}H_{20}O_{11}$	Kaempferol-3-O-rutinoside	Flavonoid glycoside	2
25	26.58	303.1	$C_{15}H_{10}O_7$	Quercetin	Flavonoid	2
26	27.49	287.1	$C_{15}H_{10}O_6$	Kaempferol	Flavonoid	2
27	28.36	317.1	$C_{16}H_{12}O_7$	Isorhamnetin	Flavonoid	2
28	29.25	595.1	$C_{27}H_{30}O_{15}$	Quercetin-3-rutinoside-pentoside	Flavonoid glycoside	3
30	31.87	301.1	$C_{16}H_{12}O_6$	Xanthone derivative	Xanthone	3
31	34.08	343.1	$C_{18}H_{14}O_7$	Xanthone glycoside	Xanthone glycoside	3

Peak No.	TR (min)[1]	Experimental m/z	Proposed molecular formula[2]	Tentative compound	Compound class	MSI level[3]
32	37.26	265.1	$C_{15}H_{12}O_4$	Benzophenone derivative	Benzophenone glycoside	3

Table S4. Provisional annotation of HPLC-ESI-MS (positive-ion mode) features detected in the ethanolic extract of Golden Gardenia (*G. sootepensis*) pulp plus peel (EEGPuPe).

Peak No.	TR (min)[1]	Experimental m/z	Proposed molecular formula[2]	Tentative compound	Compound class	MSI level[3]
1	2.28	127.0	$C_6H_6O_3$	Hydroxybenzoic acid derivative	Iridoid precursor	4
4	3.04	223.1	$C_8H_{10}O_7$	Loganin (aglycone)	Iridoid	3
5	3.39	241.1	$C_{10}H_{12}O_7$	Secoxyloganin (aglycone)	Iridoid	3
6	9.82	399.1	$C_{17}H_{22}O_{11}$	Geniposide	Iridoid glycoside	2
7	10.11	399.1	$C_{17}H_{22}O_{11}$	Gardenoside	Iridoid glycoside	2
8	10.48	227.1	$C_{10}H_{10}O_6$	Genipin (aglycone)	Iridoid	2
9	11.32	447.1	$C_{21}H_{22}O_{11}$	Gardenoside methyl ester	Iridoid glycoside	2
10	11.70	229.1	$C_{10}H_{12}O_6$	7-Deoxyloganin (aglycone)	Iridoid	3
11	11.73	225.1	$C_{10}H_{10}O_6$	7-Epideoxyloganin (aglycone)	Iridoid	3
12	12.06	223.1	$C_{10}H_{10}O_6$	10-Hydroxygeniposide (aglycone)	Iridoid	3
13	12.57	225.1	$C_{10}H_{10}O_6$	Geniposidic acid (aglycone)	Iridoid	3
14	13.20	209	$C_8H_8O_6$	Shanzhiside methyl ester	Iridoid	3
15	13.46	227.1	$C_{10}H_{10}O_6$	Geniposidic acid methyl ester	Iridoid	3
16	13.59	191.1	$C_8H_{10}O_5$	Quinic acid	Phenolic acid	2
17	15.04	235.1	$C_{12}H_{10}O_5$	Chlorogenic acid	Phenolic acid	2
18	17.33	193.1	$C_9H_8O_4$	Caffeic acid	Phenolic acid	2
19	18.11	310.1	$C_{15}H_{14}O_7$	Rutin (quercetin-3-O-rutinoside)	Phenolic glycoside	2
20	21.97	179.0	$C_5H_6O_4$	Ferulic acid	Phenolic acid	2
21	24.02	463.1	$C_{21}H_{20}O_{12}$	Quercetin-3-O-glucoside	Flavonoid glycoside	2
22	24.57	481.1	$C_{22}H_{22}O_{12}$	Quercetin-3-O-rutinoside	Flavonoid glycoside	2
23	25.31	449.1	$C_{21}H_{20}O_{11}$	Kaempferol-3-O-glucoside	Flavonoid glycoside	2
24	25.76	433.1	$C_{20}H_{20}O_{11}$	Kaempferol-3-O-rutinoside	Flavonoid glycoside	2
25	26.58	303.1	$C_{15}H_{10}O_7$	Quercetin	Flavonoid	2
26	27.49	287.1	$C_{15}H_{10}O_6$	Kaempferol	Flavonoid	2
27	28.36	317.1	$C_{16}H_{12}O_7$	Isorhamnetin	Flavonoid	2

Peak No.	T_R (min)[1]	Experimental m/z	Proposed molecular formula[2]	Tentative compound	Compound class	MSI level[3]
28	29.25	595.1	$C_{27}H_{30}O_{15}$	Quercetin-3-rutinoside-pentoside	Flavonoid glycoside	3
29	30.12	609.1	$C_{28}H_{32}O_{15}$	Acetylated hyperosides	Flavonoid glycoside	3
30	31.87	301.1	$C_{16}H_{12}O_6$	Xanthone derivative	Xanthone	3
31	34.08	343.1	$C_{18}H_{14}O_7$	Xanthone glycoside	Xanthone glycoside	3
32	37.26	265.1	$C_{15}H_{12}O_4$	Benzophenone derivative	Benzophenone glycoside	3
33	44.72	515.1	$C_{25}H_{26}O_{12}$	Iridoid–phenolic conjugate	Iridoid–conjugate	3
34	55.83	676.2	$C_{33}H_{40}O_{15}$	HMW polyphenolic conjugate	Polyphenol conjugate	4

Table S5. Provisional annotation of HPLC-ESI-MS (positive-ion mode) features detected in the water extract of Golden Gardenia (*G. sootepensis*) seeds (WEGS).

Peak No.	T_R (min)[1]	Experimental m/z	Proposed molecular formula[2]	Tentative compound	Compound class	MSI level[3]
1	2.28	127	$C_6H_6O_3$	Hydroxybenzoic acid derivative	Iridoid precursor	4
2	2.52	210.1	$C_7H_{10}O_7$	Gluconic acid	Iridoid precursor	4
3	2.82	209	$C_7H_9O_7$	Dehydroascorbic acid	Iridoid precursor	4
4	3.04	223.1	$C_8H_{10}O_7$	Loganin (aglycone)	Iridoid	3
5	3.39	241.1	$C_{10}H_{12}O_7$	Secoxyloganin (aglycone)	Iridoid	3
6	9.82	399.1	$C_{17}H_{22}O_{11}$	Geniposide	Iridoid glycoside	2
7	10.11	399.1	$C_{17}H_{22}O_{11}$	Gardenoside	Iridoid glycoside	2
8	10.48	227.1	$C_{10}H_{10}O_6$	Genipin (aglycone)	Iridoid	2
9	11.32	447.1	$C_{21}H_{22}O_{11}$	Gardenoside methyl ester	Iridoid glycoside	2
10	11.70	229.1	$C_{10}H_{12}O_6$	7-Deoxyloganin (aglycone)	Iridoid	3
11	11.73	225.1	$C_{10}H_{10}O_6$	7-Epideoxyloganin (aglycone)	Iridoid	3
12	12.06	223.1	$C_{10}H_{10}O_6$	10-Hydroxygeniposide (aglycone)	Iridoid	3
13	12.57	225.1	$C_{10}H_{10}O_6$	Geniposidic acid (aglycone)	Iridoid	3
14	13.20	209	$C_8H_8O_6$	Shanzhiside methyl ester	Iridoid	3
15	13.46	227.1	$C_{10}H_{10}O_6$	Geniposidic acid methyl ester	Iridoid	3
16	13.59	191.1	$C_8H_{10}O_5$	Quinic acid	Phenolic acid	2
17	15.04	235.1	$C_{12}H_{10}O_5$	Chlorogenic acid	Phenolic acid	2
18	17.33	193.1	$C_9H_8O_4$	Caffeic acid	Phenolic acid	2
19	18.11	310.1	$C_{15}H_{14}O_7$	Rutin (quercetin-3-O-rutinoside)	Phenolic glycoside	2
20	21.97	179	$C_5H_6O_4$	Ferulic acid	Phenolic acid	2
21	24.02	463.1	$C_{21}H_{20}O_{12}$	Quercetin-3-O-glucoside	Flavonoid glycoside	2
22	24.57	481.1	$C_{22}H_{22}O_{12}$	Quercetin-3-O-rutinoside	Flavonoid glycoside	2
23	25.31	449.1	$C_{21}H_{20}O_{11}$	Kaempferol-3-O-glucoside	Flavonoid glycoside	2
24	25.76	433.1	$C_{20}H_{20}O_{11}$	Kaempferol-3-O-rutinoside	Flavonoid glycoside	2
25	26.58	303.1	$C_{15}H_{10}O_7$	Quercetin	Flavonoid	2
26	27.49	287.1	$C_{15}H_{10}O_6$	Kaempferol	Flavonoid	2
27	28.36	317.1	$C_{16}H_{12}O_7$	Isorhamnetin	Flavonoid	2
28	29.25	595.1	$C_{27}H_{30}O_{15}$	Quercetin-3-rutinoside-pentoside	Flavonoid glycoside	3
30	31.87	301.1	$C_{16}H_{12}O_6$	Xanthone derivative	Xanthone	3

Peak No.	T_R (min)[1]	Experimental m/z	Proposed molecular formula[2]	Tentative compound	Compound class	MSI level[3]
31	34.08	343.1	$C_{18}H_{14}O_7$	Xanthone glycoside	Xanthone glycoside	3
32	37.26	265.1	$C_{15}H_{12}O_4$	Benzophenone derivative	Benzophenone glycoside	3

Table S6. Provisional annotation of HPLC-ESI-MS (positive-ion mode) features detected in the ethanolic extract of Golden Gardenia (*G. sootepensis*) seeds (EEGS).

Peak No.	T_R (min)[1]	Experimental m/z	Proposed molecular formula[2]	Tentative compound	Compound class	MSI level[3]
1	2.28	127.0	$C_6H_6O_3$	Hydroxybenzoic acid derivative	Iridoid precursor	4
2	2.52	210.1	$C_7H_{10}O_7$	Gluconic acid	Iridoid precursor	4
3	2.82	209.0	$C_7H_9O_7$	Dehydroascorbic acid	Iridoid precursor	4
4	3.04	223.1	$C_8H_{10}O_7$	Loganin (aglycone)	Iridoid	3
5	3.39	241.1	$C_{10}H_{12}O_7$	Secoxyloganin (aglycone)	Iridoid	3
6	9.82	399.1	$C_{17}H_{22}O_{11}$	Geniposide	Iridoid glycoside	2
7	10.11	399.1	$C_{17}H_{22}O_{11}$	Gardenoside	Iridoid glycoside	2
8	10.48	227.1	$C_{10}H_{10}O_6$	Genipin (aglycone)	Iridoid	2
9	11.32	447.1	$C_{21}H_{22}O_{11}$	Gardenoside methyl ester	Iridoid glycoside	2
10	11.70	229.1	$C_{10}H_{12}O_6$	7-Deoxyloganin (aglycone)	Iridoid	3
11	11.73	225.1	$C_{10}H_{10}O_6$	7-Epideoxyloganin (aglycone)	Iridoid	3
12	12.06	223.1	$C_{10}H_{10}O_6$	10-Hydroxygeniposide (aglycone)	Iridoid	3
13	12.57	225.1	$C_{10}H_{10}O_6$	Geniposidic acid (aglycone)	Iridoid	3
14	13.20	209.0	$C_8H_8O_6$	Shanzhiside methyl ester	Iridoid	3
15	13.46	227.1	$C_{10}H_{10}O_6$	Geniposidic acid methyl ester	Iridoid	3
16	13.59	191.1	$C_8H_{10}O_5$	Quinic acid	Phenolic acid	2
17	15.04	235.1	$C_{12}H_{10}O_5$	Chlorogenic acid	Phenolic acid	2
18	17.33	193.1	$C_9H_8O_4$	Caffeic acid	Phenolic acid	2
19	18.11	310.1	$C_{15}H_{14}O_7$	Rutin (quercetin-3-O-rutinoside)	Phenolic glycoside	2
20	21.97	179.0	$C_5H_6O_4$	Ferulic acid	Phenolic acid	2
21	24.02	463.1	$C_{21}H_{20}O_{12}$	Quercetin-3-O-glucoside	Flavonoid glycoside	2
22	24.57	481.1	$C_{22}H_{22}O_{12}$	Quercetin-3-O-rutinoside	Flavonoid glycoside	2
23	25.31	449.1	$C_{21}H_{20}O_{11}$	Kaempferol-3-O-glucoside	Flavonoid glycoside	2
24	25.76	433.1	$C_{20}H_{20}O_{11}$	Kaempferol-3-O-rutinoside	Flavonoid glycoside	2
25	26.58	303.1	$C_{15}H_{10}O_7$	Quercetin	Flavonoid	2
26	27.49	287.1	$C_{15}H_{10}O_6$	Kaempferol	Flavonoid	2
27	28.36	317.1	$C_{16}H_{12}O_7$	Isorhamnetin	Flavonoid	2
28	29.25	595.1	$C_{27}H_{30}O_{15}$	Quercetin-3-rutinoside-pentoside	Flavonoid glycoside	3
29	30.12	609.1	$C_{28}H_{32}O_{15}$	Acetylated hyperosides	Flavonoid glycoside	3

Peak No.	T_R (min)[1]	Experimental m/z	Proposed molecular formula[2]	Tentative compound	Compound class	MSI level[3]
30	31.87	301.1	$C_{16}H_{12}O_6$	Xanthone derivative	Xanthone	3
31	34.08	343.1	$C_{18}H_{14}O_7$	Xanthone glycoside	Xanthone glycoside	3
32	37.26	265.1	$C_{15}H_{12}O_4$	Benzophenone derivative	Benzophenone glycoside	3
33	44.72	515.1	$C_{25}H_{26}O_{12}$	Iridoid–phenolic conjugate	Iridoid–conjugate	3
34	55.83	676.2	$C_{33}H_{40}O_{15}$	HMW polyphenolic conjugate	Polyphenol conjugate	4

Table S7. Comparative distribution of provisionally annotated HPLC-ESI-MS (positive-ion mode) phytochemical features across WEGL, EEGL, WEGPuPe, EEGPuPe, WEGS, and EEGS.

Peak No.	T_R (min)[1]	Experimental m/z	Proposed molecular formula[2]	Tentative compound	Compound class	MSI level[3]	WEGL	EEGL	WEGPuPe	EEGPuPe	WEGS	EE[illegible]
1	2.28	127	$C_6H_6O_3$	Hydroxybenzoic acid derivative	Iridoid precursor	4	✓	✓	✓	✓	✓	✓
2	2.52	210.1	$C_7H_{10}O_7$	Gluconic acid	Iridoid precursor	4	✓	✓	–	–	✓	✓
3	2.82	209	$C_7H_9O_7$	Dehydroascorbic acid	Iridoid precursor	4	✓	✓	–	–	✓	✓
4	3.04	223.1	$C_8H_{10}O_7$	Loganin (aglycone)	Iridoid	3	✓	✓	✓	✓	✓	✓
5	3.39	241.1	$C_{10}H_{12}O_7$	Secoxyloganin (aglycone)	Iridoid	3	✓	✓	✓	✓	✓	✓
6	9.82	399.1	$C_{17}H_{22}O_{11}$	Geniposide	Iridoid glycoside	2	✓	✓	✓	✓	✓	✓
7	10.11	399.1	$C_{17}H_{22}O_{11}$	Gardenoside	Iridoid glycoside	2	✓	✓	✓	✓	✓	✓
8	10.48	227.1	$C_{10}H_{10}O_6$	Genipin (aglycone)	Iridoid	2	✓	✓	✓	✓	✓	✓
9	11.32	447.1	$C_{21}H_{22}O_{11}$	Gardenoside methyl ester	Iridoid glycoside	2	✓	✓	✓	✓	✓	✓
10	11.70	229.1	$C_{10}H_{12}O_6$	7-Deoxyloganin (aglycone)	Iridoid	3	✓	✓	✓	✓	✓	✓
11	11.73	225.1	$C_{10}H_{10}O_6$	7-Epideoxyloganin (aglycone)	Iridoid	3	✓	✓	✓	✓	✓	✓
12	12.06	223.1	$C_{10}H_{10}O_6$	10-Hydroxygeniposide (aglycone)	Iridoid	3	✓	✓	✓	✓	✓	✓
13	12.57	225.1	$C_{10}H_{10}O_6$	Geniposidic acid (aglycone)	Iridoid	3	✓	✓	✓	✓	✓	✓
14	13.20	209	$C_8H_8O_6$	Shanzhiside methyl ester	Iridoid	3	✓	✓	✓	✓	✓	✓
15	13.46	227.1	$C_{10}H_{10}O_6$	Geniposidic acid methyl ester	Iridoid	3	✓	✓	✓	✓	✓	✓
16	13.59	191.1	$C_8H_{10}O_5$	Quinic acid	Phenolic acid	2	✓	✓	✓	✓	✓	✓
17	15.04	235.1	$C_{12}H_{10}O_5$	Chlorogenic acid	Phenolic acid	2	✓	✓	✓	✓	✓	✓
18	17.33	193.1	$C_9H_8O_4$	Caffeic acid	Phenolic acid	2	✓	✓	✓	✓	✓	✓
19	18.11	310.1	$C_{15}H_{14}O_7$	Rutin (quercetin-3-O-rutinoside)	Phenolic glycoside	2	✓	✓	✓	✓	✓	✓
20	21.97	179	$C_5H_6O_4$	Ferulic acid	Phenolic acid	2	✓	✓	✓	✓	✓	✓

Peak No.	T_R (min)[1]	Experimental m/z	Proposed molecular formula[2]	Tentative compound	Compound class	MSI level[3]	WEGL	EEGL	WEGPuPe	EEGPuPe	WEGS	EEG[illegible]
21	24.02	463.1	$C_{21}H_{20}O_{12}$	Quercetin-3-O-glucoside	Flavonoid glycoside	2	✓	✓	✓	✓	✓	✓
22	24.57	481.1	$C_{22}H_{22}O_{12}$	Quercetin-3-O-rutinoside	Flavonoid glycoside	2	✓	✓	✓	✓	✓	✓
23	25.31	449.1	$C_{21}H_{20}O_{11}$	Kaempferol-3-O-glucoside	Flavonoid glycoside	2	✓	✓	✓	✓	✓	✓
24	25.76	433.1	$C_{20}H_{20}O_{11}$	Kaempferol-3-O-rutinoside	Flavonoid glycoside	2	✓	✓	✓	✓	✓	✓
25	26.58	303.1	$C_{15}H_{10}O_7$	Quercetin	Flavonoid	2	✓	✓	✓	✓	✓	✓
26	27.49	287.1	$C_{15}H_{10}O_6$	Kaempferol	Flavonoid	2	✓	✓	✓	✓	✓	✓
27	28.36	317.1	$C_{16}H_{12}O_7$	Isorhamnetin	Flavonoid	2	✓	✓	✓	✓	✓	✓
28	29.25	595.1	$C_{27}H_{30}O_{15}$	Quercetin-3-rutinoside-pentoside	Flavonoid glycoside	3	✓	✓	✓	✓	✓	✓
29	30.12	609.1	$C_{28}H_{32}O_{15}$	Acetylated hyperosides	Flavonoid glycoside	3	–	✓	–	✓	–	✓
30	31.87	301.1	$C_{16}H_{12}O_6$	Xanthone derivative	Xanthone	3	✓	✓	✓	✓	✓	✓
31	34.08	343.1	$C_{18}H_{14}O_7$	Xanthone glycoside	Xanthone glycoside	3	✓	✓	✓	✓	✓	✓
32	37.26	265.1	$C_{15}H_{12}O_4$	Benzophenone derivative	Benzophenone glycoside	3	✓	✓	✓	✓	✓	✓
33	44.72	515.1	$C_{25}H_{26}O_{12}$	Iridoid–phenolic conjugate	Iridoid–conjugate	3	–	✓	–	✓	–	✓
34	55.83	676.2	$C_{33}H_{40}O_{15}$	HMW polyphenolic conjugate	Polyphenol conjugate	4	–	✓	–	✓	–	✓
